# Pathologically high intraocular pressure disturbs normal iron homeostasis and leads to retinal ganglion cell ferroptosis in glaucoma

**DOI:** 10.1038/s41418-022-01046-4

**Published:** 2022-08-06

**Authors:** Fei Yao, Jingjie Peng, Endong Zhang, Dan Ji, Zhaolin Gao, Yixiong Tang, Xueyan Yao, Xiaobo Xia

**Affiliations:** 1 Eye Center of Xiangya Hospital, Central South University, Changsha, Hunan China; 2 Hunan Key Laboratory of Ophthalmology, Changsha, Hunan China; 3 National Clinical Research Center for Geriatric Disorders, Xiangya Hospital, Changsha, Hunan China

**Keywords:** Neurological disorders, Pathogenesis, Peripheral nervous system, Metals

## Abstract

Glaucoma can result in retinal ganglion cell (RGC) death and permanently damaged vision. Pathologically high intraocular pressure (ph-IOP) is the leading cause of damaged vision during glaucoma; however, controlling ph-IOP alone does not entirely prevent the loss of glaucomatous RGCs, and the underlying mechanism remains elusive. In this study, we reported an increase in ferric iron in patients with acute primary angle-closure glaucoma (the most typical glaucoma with ph-IOP damage) compared with the average population by analyzing free iron levels in peripheral serum. Thus, iron metabolism might be involved in regulating the injury of RGCs under ph-IOP. In vitro and in vivo studies confirmed that ph-IOP led to abnormal accumulation of ferrous iron in cells and retinas at 1–8 h post-injury and elevation of ferric iron in serum at 8 h post-injury. Nuclear receptor coactivator 4 (NCOA4)-mediated degradation of ferritin heavy polypeptide 1(FTH1) is essential to disrupt iron metabolism in the retina after ph-IOP injury. Furthermore, knockdown of *Ncoa4* in vivo inhibited FTH1 degradation and reduced the retinal ferrous iron level. Elevated ferrous iron induced by ph-IOP led to a marked accumulation of pro-ferroptotic factors (lipid peroxidation and acyl CoA synthetase long-chain family member 4) and a depletion of anti-ferroptotic factors (glutathione, glutathione peroxidase 4, and nicotinamide adenine dinucleotide phosphate). These biochemical changes resulted in RGC ferroptosis. Deferiprone can pass through the blood-retinal barrier after oral administration and chelated abnormally elevated ferrous iron in the retina after ph-IOP injury, thus inhibiting RGC ferroptosis and protecting visual function. In conclusion, this study revealed the role of NCOA4-FTH1-mediated disturbance of iron metabolism and ferroptosis in RGCs during glaucoma. We demonstrate the protective effect of Deferiprone on RGCs *via* inhibition of ferroptosis, providing a research direction to understand and treat glaucoma *via* the iron homeostasis and ferroptosis pathways.

## Introduction

Glaucoma is the leading cause of irreversible blinding worldwide. According to the World Health Organization (WHO), approximately 76 million people suffered from glaucomatous conditions in 2020 [1]. These numbers are estimated to exceed 11.1 million by 2040, detrimentally affecting the life quality of patients worldwide [2]. Glaucoma is characterized by progressive visual field defects, often resulting from selective and irreversible loss of retinal ganglion cells (RGCs), the only efferent retinal neurons that directly project their axons into the central nervous system and perform a visual function [3]. Based on different disease characteristics, glaucoma can be classified into primary glaucoma, secondary glaucoma, childhood glaucoma, and combined mechanism glaucoma. However, regardless of taxonomy, pathologically high intraocular pressure (ph-IOP) is the main cause of injury to glaucomatous RGCs [4].

The mechanisms underlying RGC injury during ph-IOP include: (1) Mechanical pressure on the optic disc and lamina cribrosa, which distorts the fovea, compresses RGC axons, and blocks axoplasmic transport, resulting in the activation of apoptotic and necrotic pathways; (2) decreased retinal blood perfusion, resulting in RGC ischemic injury; (3) activation of immune responses, leading to inflammatory cell infiltration that injures RGCs; and (4) other potential mechanisms [5–7]. Effective clinical options are limited, and current glaucoma treatments aim to lower ph-IOP using medications or surgery [8]. Unfortunately, lowering ph-IOP does not entirely prevent glaucoma progression or the damage to RGCs [9]. A reduced visual field persist in many patients with glaucoma even after ph-IOP is controlled to normal levels. Clearly, other pathogenic factors are involved in the process of RGC damage after IOP restoration [10, 11]. Finding and controlling these causative factors are essential to manage and improve glaucoma prognosis [12].

Iron is an essential element in the human body and is involved in the biosynthesis of DNA, RNA, and proteins, playing a pivotal role in energy metabolism, the cell cycle, growth, and development processes [13]. In the eye, abnormal iron metabolism affects the pathological process of multiple ophthalmic diseases [14, 15]. For example, abnormal iron accumulation in the retina promotes the development of age-related maculopathy, and iron accumulation in the intravitreal or intravitreal subretinal effusion is closely related to poor visual recovery after retinal detachment [14, 16]. Some epidemiological studies show that a high iron diet and elevated serum ferritin are associated positively with increased glaucoma incidence, suggesting that iron metabolism is related to glaucoma pathogenesis [17, 18]. However, it remains unclear how iron metabolism specifically affects glaucoma. The concept of ferroptosis (proposed in 2012) provided us with a new approach to determine the pathogenesis of glaucoma from the perspective of iron metabolism.

Ferroptosis is a form of iron-regulated cell death, which is distinguished from other traditional cell death programs by its morphological (shrunken mitochondria with increased membrane density), biochemical (accumulation of free iron and toxic lipid peroxidation, depletion of intracellular antioxidants), and genetic (involvement of many specific genes) features [19]. Ferroptosis participates in numerous pathophysiological processes and is a promising therapeutic target for human diseases, such as cancer, neurodegenerative diseases, and ischemic organ damage [20, 21]. However, no studies have linked ferroptosis to glaucoma. Our previous study found that deferoxamine, an ferroptosis inhibitor, could attenuate N-methyl-D-aspartate-induced RGC-5 cell damage (a glaucoma excitotoxicity model) [22], suggesting ferroptosis as a novel target for glaucoma therapy. In addition, the ischemic injury and neuronal degeneration of RGCs caused by ph-IOP have similarities to diseases associated with ferroptosis. Therefore, we speculated that ferroptosis is also involved in ph-IOP-induced glaucomatous RGC loss, and this involvement is closely regulated by iron metabolism.

We selected acute primary angle-closure glaucoma (APACG), the most typical form of ph-IOP impairment clinically, to test our hypothesis. Compared with other types of glaucoma, APACG is characterized by pronounced elevation of IOP, acute disease onset, and significant RGC loss within a short period [23]. Additionally, APACG is more prone to blood perfusion sensitivity after IOP reduction, leading to ischemia-reperfusion injury and aggravating RGC death [23, 24], making it an ideal model to study the continued loss of RGCs after IOP control in glaucoma. Specifically, we looked for clinical evidence of an imbalance in iron homeostasis after ph-IOP insult by analyzing the free iron content in the serum of patients with APACG. Cellular and animal models of ph-IOP injury were established to verify the characteristics and mechanisms of iron homeostasis imbalance. The regulation of ferroptosis by imbalanced iron homeostasis was also investigated. Finally, deferiprone, an iron chelator, was demonstrated to inhibit iron homeostasis imbalance and ferroptosis, and protect RGCs and visual function. Our results revealed new targets to treat glaucomatous RGC loss.

## Materials and methods

### Animals

Male C57BL/6 mice (8 weeks old; Slaccas, Changsha, China) were randomly divided into groups and reared with free access to water and food under a 12-h light-dark cycle. In all procedures, the mice were anesthetized using a solution of 1% sodium pentobarbital (100 mg/kg, i.p.; Sanshu, Beijing, China) and 10 mg/kg xylazine (Huamu, Beijing, China). Oxybuprocaine hydrochloride and tropicamide phenylephrine (both Santen Pharmaceuticals, Tokyo, Japan) were applied to anesthetize the mice’s corneas and dilate their pupils. All animal experiments were carried out in accordance with the *Guide for the Care and Use of Laboratory Animals* (National Institutes of Health, Bethesda, MD, USA). The study protocols were approved by the Animal Research Committee of the Xiangya School of Medicine (approval number: 2019sydw0201; Changsha, China).

### Model of ph-IOP injury

**Fig. 1:**
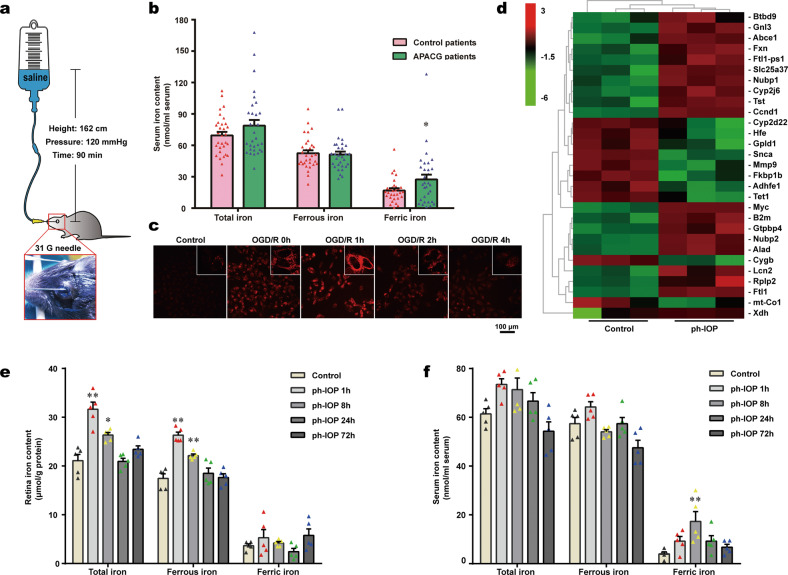
Pathologically high intraocular pressure (ph-IOP) injury disturbed normal iron homeostasis. **a** diagram of ph-IOP injury modeling. **b** Serum iron contents in patients with acute primary angle-closure glaucoma (APACG) and healthy controls (*n* = 31 in each group). **c** Cytoplasmic ferrous iron stained using FerroOrange in R28 cells (red) treated with oxygen-glucose deprivation/ reoxygenation (OGD/R). **d** Full-length transcriptome analysis showing the transcription levels of 29 iron metabolism-related genes in ph-IOP injured mice and control mice at 24 h after modeling. Comparison of the iron content in the retina (**e**) and serum (**f**) between control mice and ph-IOP injured mice (*n* = 5 in each group). Data are shown as the mean ± SD; *p < 0.05, **p < 0.01 (compared with the control group using one-way analysis of variance). Bar = 100 μm.

Ph-IOP injury was induced by increasing the anterior chamber pressure *via* a saline-perfusion system described by Matthew et al. [25]. Briefly, an anesthetized mouse with dilated pupils and anesthetized corneas was placed on a heating workbench. A 31G needle connected to a saline infusion set was carefully inserted into the mouse’s anterior chamber and the anterior chamber pressure was gradually increased to 120 mmHg by adjusting saline bottle height (162 cm); the pressure was maintained for 90 min (Fig. 1a). Eyes that only underwent needle puncture without saline pressurization were considered as sham operation (SO) eyes. During and after surgery, 0.3% tobramycin eye ointment (Alcon, Houston, TX, USA) was applied to keep the eyes moist and uninfected. Only mice without saline leakage, or lens and iris injury were included in this study.

### Cell culture and the oxygen-glucose deprivation/reoxygenation (OGD/R) model

Retinal cell line R28, an immortalized adherent retinal precursor cell line derived from infantile Sprague-Dawley rat retinas, is commonly used for in vitro studies of the neuroprotection and physiological function of RGCs [26, 27]. The Department of Anatomy and Neurobiology (Central South University, Changsha, China) provided the R28 cells. The R28 culture medium comprised low glucose Dulbecco’s modified Eagle’s medium (DMEM; 11885084; Gibco, Carlsbad, CA, USA), 10% fetal calf serum (0500; ScienCell, San Diego, CA, USA), 1% non-essential amino acids (M7145; Sigma, St. Louis, MO, USA), 1% penicillin-streptomycin (60162ES76; Yeasen, Shanghai, China), and 1% L-glutamine (G3126; Sigma). We established the R28 cell OGD/R model as follows: The original cell culture medium was replaced with glucose-free DMEM (11966025; Gibco) and the R28 cells were incubated in an anoxic environment (95% N_2_, 5% CO_2_) for 2 h. The medium was changed back to the original medium and R28 cells were reoxygenated (21% O_2_, 5% CO_2_) for 0, 1, 2, and 4 h until examined.

### Deferiprone treatment

In vivo, the oral iron chelator Deferiprone (DFP, 0652-11-0; ACROS ORGANICS, Newark, NJ, USA) was added to the mice’s daily drinking water at 1 mg/ml (according to a previous study [28]) 1 day before modeling; the DFP drinking water was renewed every other day. The mice were given 200 mg/kg supplementary DFP treatment by oral gavage 30 min before modeling. In vitro, R28 cells were pretreated with 1 mM DFP (according to a previous study [29]) 1 h before and during OGD/R modeling.

### Acquisition of patient serum

We collected 1.5 ml of serum from inpatients with APACG and isometric serum from non-glaucomatous outpatients in the Xiangya Hospital. The patients and controls provided written informed consent. The procedures were approved by the ethics committee of Xiangya Hospital (IRB approval number: 202008100), and were performed according to the tenets of the Declaration of Helsinki. Freshly collected serum was frozen immediately and stored at −80 °C until analysis.

### Measurement of retinal oxidative stress

Malondialdehyde (MDA), the reduced form of nicotinamide adenine dinucleotide phosphate (NADPH), and glutathione (GSH) are crucial biochemical indicators reflecting oxidative stress levels [30–32]. The retinal MDA content was measured using a Lipid Peroxidation MDA Assay Kit (S0131S; Beyotime, Shanghai, China). The retinal NADPH continent was measured using an NADP/NADPH Assay Kit (ab65349; Abcam, Cambridge, UK). The retinal GSH content was measured using a Micro Reduced GSH Assay Kit (BC1175; Solarbio, Beijing, China). The measured contents were calibrated using the samples’ protein concentration.

### High Performance Liquid Chromatography (HPLC)

HPLC was used to detect the DFP concentration in mouse serum and retinas after 200 mg/kg DFP treatment by oral gavage. Briefly, mice were sacrificed 1 h after DFP administration, and their serum and retinas were collected. The collected samples and a DFP standard (30652-11-0; Wanjia Biotechnology, Beijing, China) were prepared for HPLC detection (1260 Infinity II; Agilent, Santa Clara, CA, USA) according to a standard protocol. The chromatograms were recorded and analyzed using ChemStation software (Agilent). Serum DFP concentrations were calibrated by the sample volume. The retinal DFP concentration was calibrated by the sample weight.

### Full-length transcriptome analysis

Mouse retinas were collected 24 h after ph-IOP injury or SO induction. Four individual retinas were pooled and treated as one sample; each group contained three samples. Total RNA was isolated using TRIzol (Invitrogen, Carlsbad, CA, USA) according to the manufacturer’s instructions, then 1 µg of total RNA was prepared for cDNA library construction using a cDNA-PCR Sequencing Kit (SQK-PCS109, Oxford Nanopore Technologies Ltd., Oxford, UK). The final cDNA libraries were added to FLO-MIN109 flow cells and run on the PromethION platform (both Oxford Nanopore Technologies Ltd.) at the Biomarker Technology Company (Beijing, China). Genes with a false discovery rate (FDR) < 0.05 and a fold-change ≥1.5 identified by DESeq2 were considered differentially expressed [33]. Gene functional annotations were assessed based on the following databases: NR (NCBI non-redundant protein sequences); Pfam (protein family); KOG/COG/eggNOG (Clusters of Orthologous Groups of proteins); Swiss-Prot (a protein sequence database); KEGG (Kyoto Encyclopedia of Genes and Genomes), and GO (Gene Ontology).

### Retina morphological analysis

Anesthetized mice were perfused transcardially with pre-cooled saline. Intact eyes were collected and fixed in FAS eyeball fixative solution (G1109; Servicebio, Wuhan, China) overnight at 4 °C. Fixed eyes were then dehydrated, embedded in paraffin, and sectioned at 3 µm thickness around the optic nerve.

Hematoxylin and eosin (H&E) staining of retinal sections was performed using a Hematoxylin-Eosin staining Kit (G1005; Servicebio). To better represent the morphological changes in the retina, 12 points of retinal thickness and ganglion cell complex (GCC, consisting of a retinal nerve fiber layer, a ganglion cell layer, and an inner plexiform layer, which corresponds exactly to the anatomical distribution of RGCs in the retina [34]) thickness per retinal slice were measured using Image-Pro Plus 6.0 software (Media Cybernetics, Rockville, MD, USA) with the following parameters: Perpendicular to the retinal pigment epithelium layer as well as ±360, ±720, ±1080, ±1440, ±1800, and ±2160 μm away from the center of the optic nerve (Supplemental Fig. S1).

Retinal immunofluorescence (IF) staining was carried out as described previously [35]. Four antibodies were used: Anti-acyl-CoA synthetase long-chain family member 4 (ACSL4; ab155282; Abcam; 1:150), anti-glutathione peroxidase 4 (GPX4; ab125066; Abcam; 1:100), anti-ferritin heavy polypeptide 1 (FTH1; ab65080; Abcam; 1:200), and anti-RNA-binding protein with multiple splicing (RBPMs; 1832-RBPMS; PhosphoSolutions, Aurora, CO, USA; 1:500).

### Iron parameters

Serum and retinal iron levels (total iron, ferrous iron, and ferric iron) were measured using an Iron Assay Kit (MAK025; Sigma) according to the manufacturer’s instructions. Perl’s staining was used to detect the ferric iron distribution in each layer of the retina. Briefly, deparaffinized and rehydrated retinal paraffin sections (3 µm) were stained using a Prussian blue staining kit (G1029; Servicebio) according to the recommended protocol. FerroOrange (F374; Dojindo, Beijing, China) was applied to reveal the distribution and expression of ferrous iron in the cytoplasm of living R28 cells. Briefly, the R28 cell culture medium was replaced with serum-free medium containing 1 μM FerroOrange, and then the R28 cells were incubated at 37 °C with an atmosphere containing 5% CO_2_ for 30 min before being detected using a confocal fluorescence microscope (Leica, Frankfurt, Germany).

### Western blotting

Freshly isolated mouse retinas were homogenized and lysed in ice-cold Radioimmunoprecipitation assay (RIPA) buffer (P0013; Beyotime). A Bicinchoninic Acid Kit (Pierce, Rockford, IL, USA) was used to quantify the total protein content. Western blotting was performed as described in our previous study [35] using the following antibodies: Anti-ACSL4 (as used in IF; 1:3000), anti-GPX4 (as used in IF; 1:1000), anti-FTH1 (as used in IF; 1:1000), anti-divalent metal transporter 1 (DMT1; ab55735; Abcam; 1:1000), anti-transferrin receptor (TR; ab84036; Abcam; 1:1000), anti-ferroportin 1 (Fpn1; ab235166; Abcam; 1:500), anti-nuclear receptor coactivator 4 (NCOA4; sc-373739; Santa Cruz Biotechnology, Santa Cruz, CA, USA; 1:500), and anti-β-actin (GB12001; Servicebio; 1:3000). The original western blots for these results are provided in Supplementary File 1.

### Co-immunoprecipitation (Co-IP)

Freshly collected retinas were treated and lysed with IP lysis buffer (G2038; Servicebio) containing protease inhibitor cocktail (G2006; Servicebio). The tissue lysates were incubated with 1 μg NCOA4 antibody overnight at 4 °C. Immunocomplexes were captured using 80 μl protein A/G agarose mixture (IP05; Millipore, MA, USA) for 3 h at 4 °C and then washed three times in cold PBS supplemented with protease inhibitor cocktail. Eluted immunocomplexes were subjected to SDS-PAGE (G2003; Servicebio) and immunoblotted with NCOA4 and FTH1 antibodies as described in Western blotting.

### Retrograde tracing of RGCs

Three days before sacrifice, mice were anesthetized and fixed in a stereotaxic instrument (RWD Life Science, Shenzhen, China) using blunt ear bars. Then, 0.5 μl of 4% fluorogold solution (FG; Fluorochrome, Denver, CO, USA) was slowly injected into the bilateral superior colliculi, separately (4.0 mm posterior to the bregma, 1.0 mm lateral to the cranial midline, and 1.5 mm deep to the cranial surface), as described previously [36]. Three days later, the FG retrograde-labeled retinas were harvested and flattened on microscope slides. A fluorescence microscope (DM5000 B; Leica, Wetzlar, Germany) was used for FG (+) RGC observation. Eight images per retina were taken at 0.85 and 1.71 mm (central retina and peripheral retina) from the optic disk in the superotemporal, superonasal, inferonasal, and inferotemporal quadrants. Image-Pro Plus 6.0 software was used to count the FG-labeled RGCs in each photomicrograph.

### Optical coherence tomography (OCT)

OCT was performed using a Retinal Imaging Microscope (Micron IV; Phoenix Research Lab, Pleasanton, CA, USA) for the comprehensive evaluation of in vivo retinal leak (abnormal yellow reflection in the fundus or locally ridged bright spots within the retina). Retinal non-leaking area were recorded and analyzed using Image-Pro Plus 6.0 software.

### Flash visual-evoked potentials (FVEPs)

Retinal functional variations after modeling and DFP treatment were evaluated using a multifocal electroretinography recorder (GT-2008V-VI; Gotec, Chongqing, China) by analyzing the latency of the first positive wave (P1) and second positive wave (P2) of FVEPs. The light stimulation intensity was 10.0 cd·s/m^2^, the stimulation frequency was 1 Hz, and the stimulation number was 64. After light adaptation for 30 min, the anesthetized mouse was placed on a flat holder with three silver electrodes inserted under the skin of the occipital bone (anode), anterior bregma (cathode), and ear (ground electrode), respectively. After preparation, the FVEPs waves of the right and left eyes were recorded using a Ganzfeld electrodiagnostic analysis system (Gotec), and the latency of the P1 and P2 waves were measured.

### Transmission electron microscopy (TEM)

Freshly enucleated eyeballs were immediately put into precooled 2.5% glutaraldehyde. The corneas, lens, and vitreous humor were carefully removed in the glutaraldehyde solution, and the retinas were completely separated and fixed in a new glutaraldehyde solution for 24 h at 4 °C. Then, the fixed retinas were processed for TEM imaging following a standard protocol. The mitochondria of RGCs were viewed under a HT7700 transmission electron microscope (Hitachi, Tokyo, Japan), and the mitochondria with ferroptosis features were recorded.

### Adeno-associated virus (AAV) intravitreal injection

The experimental virus vectors, pAAV-U6-sh*Ncoa4*-CMV-EGFP-WPRE and pAAV-U6-spgRNA-CMV-EGFP-WPRE (mimic control) were packaged and purchased from OBiO Technology (Shanghai, China; Supplementary Table 1). For intravitreal injection, an anesthetized mouse with dilated pupils was put under a stereomicroscope, and 1 μl of 1.5 × 10^12^ viral genomes (v.g.)/ml AAV solution per eye was injected into the vitreous chamber using a 2.5-μl Hamilton syringe (Hamilton AG, Bonaduz, Switzerland). Mice were injected at 5 weeks old and analyzed at 8 weeks old.

### Statistical analysis

All the presented data were taken from distinct samples, as noted in figure legends, and no data were excluded. Sample sizes were based in standard protocols in the field, and at least three biological independent replicates were performed for each experiment. Experiments were blinded to the person performing data extraction and analysis. SPSS version 22.0 (IBM, Armonk, NY, USA) was used for the statistical analyses. Data are expressed as the mean ± standard deviation (SD). *T*-tests and one-way analysis of variance (ANOVA) followed by Tukey’s *post hoc* test were used for comparisons between two sets and more than two sets, respectively. Before assumptions for these tests, sample independence, normal distribution, and variance equality were assumed to be met. Statistical significance was set at *p* < 0.05.

## Results

### Ph-IOP injury disturbed iron homeostasis

To verify whether iron is involved in ph-IOP-induced RGC injury, we detected the serum iron content of patients with APACG and healthy controls. Serum total iron and ferric iron levels in patients with APACG were higher than those in the healthy controls (Fig. 1b). Nevertheless, the change in ferrous iron content was not significant. This result suggested that iron was likely to participate in the mechanism of ph-IOP-induced RGC injury.

To further clarify the relationship between ph-IOP and iron homeostasis, we established a corresponding model in vitro (Supplementary Fig. S2) and in vivo (Fig. 1a and Supplementary Fig. S1). OGD/R-treated R28 cells showed significant cytoplasmic accumulation of ferrous iron compared with that in the control group (Fig. 1c). Similarly, retinal full-length transcriptome analysis showed that the transcript levels of 29 iron metabolism-related genes changed significantly after ph-IOP injury (Fig. 1d). Moreover, both retinal and serum total iron levels increased at 1 and 8 h after ph-IOP injury compared with that in normal mice (Fig. 1e, f and Supplementary Table 2). Interestingly, increased retinal free iron was dominated by ferrous iron (the ferric iron level was unchanged), while serum was dominated by ferric iron (the ferrous iron level did not change significantly) (Fig. 1e, f and Supplementary Table 2). Prussian blue staining also indicated that the retinal ferric iron level did not change after ph-IOP injury (Supplementary Fig. S3). These results suggested that ph-IOP disturbed normal iron homeostasis, leading to significant iron accumulation in vivo and in vitro, which occurred mainly at the early stage after ph-IOP injury.

### NCOA4-mediated FTH1 degradation led to ph-IOP-induced iron accumulation

**Fig. 2:**
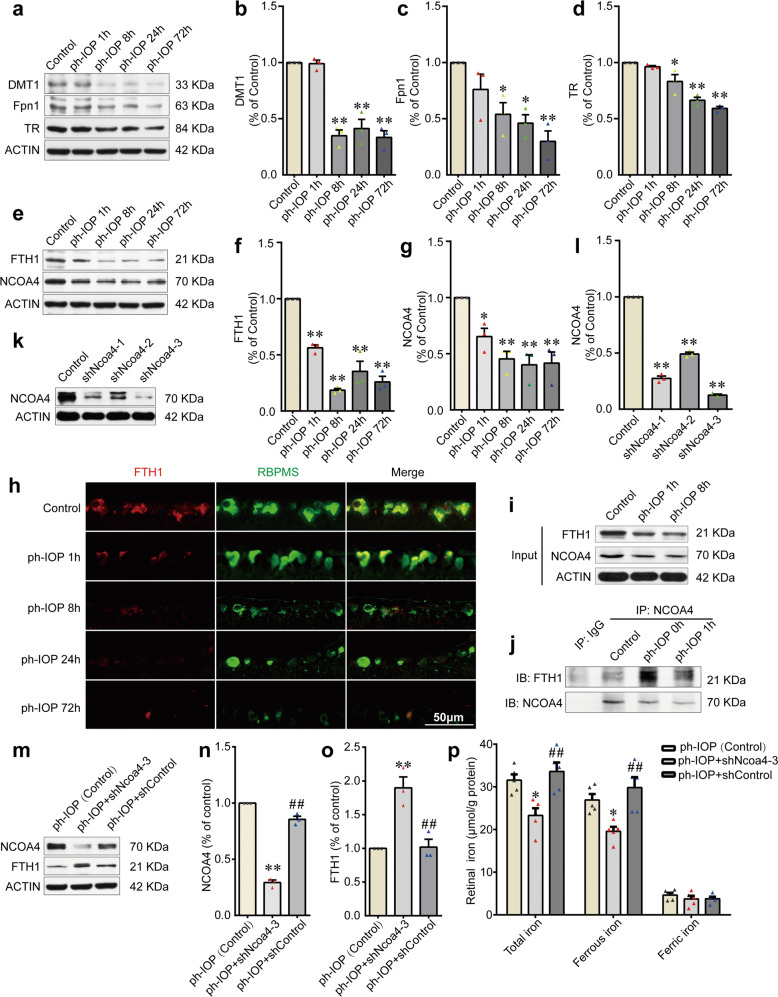
NCOA4-mediated FTH1 degradation led to pathologically high intraocular pressure (ph-IOP)-induced iron accumulation in retinas. **a–d** Western blotting detection of the retinal levels of DMT1, Fpn1, and TR (normalized to that of actin) in control and ph-IOP injured mice (*n* = 3 in each group). **e**–**g** Western blotting detection of the retinal levels of FTH1 and NCOA4 (normalized to that of actin) in control and ph-IOP injured mice (*n* = 3 in each group). **h** Representative photomicrographs of immunofluorescence staining for FTH1 (red) in retinal ganglion cells (counterstained with RBPMS; green). **I**, **j** Co-immunoprecipitation showing the endogenous interaction between NCOA4 and FTH1 in control and ph-IOP injured mice. **k**, **l** Western blotting detection of the retinal levels of NCOA4 (normalized to that of actin) in mice receiving pAAV-spgRNA-EGFP (control) and pAAV-sh*Ncoa4*-EGFP injection for 3 weeks (n = 3 in each group). **m**–**o** Western blotting detection of the retinal levels of NCOA4 and FTH1 (normalized to that of actin) in ph-IOP injured mice, with or without AAV-mediated *Ncoa4* knockdown at 1 h after modeling (*n* = 3 in each group). **p** Retinal iron contents in ph-IOP injured mice, with or without AAV-mediated *Ncoa4* knockdown, at 1 h after modeling (*n* = 5 in each group). DMT1, divalent metal transporter 1; Fpn1, ferroportin 1; FTH1, ferritin heavy polypeptide 1; NCOA4, nuclear receptor coactivator 4; TR, transferrin receptor. Data are shown as the mean ± SD; *p < 0.05, **p < 0.01 (compared with the control group using one-way analysis of variance); ##*p* < 0.01 (compared with the sh*Ncoa4* group using one-way analysis of variance). Bar = 50 μm.

To clarify how ph-IOP disrupts retinal iron homeostasis, we examined the retinal levels of several iron metabolism-related proteins after ph-IOP injury at different time points. Proteins involved in cellular iron uptake (DMT1 and TR) and iron export (Fpn1) were downregulated over time after modeling (Fig. 2a–d and Supplementary Table 2). These results indicated that retinal iron increase was not caused by excessive iron intake nor by decreased iron excretion, because Fpn1 levels changed markedly later than the change in retinal the iron level (Fig. 1e and Fig. 2a, c and Supplementary Table 2). The most likely cause of the rapid accumulation of iron in the retina after ph-IOP injury was free iron release from endogenous bound iron. Ferritin is the main intracellular iron storage protein. The level of FTH1 (a subunit of ferritin) decreased sharply at 1 h after ph-IOP injury (Fig. 2e, f, h and Supplementary Table 2), which was consistent with the iron accumulation in the early phase after ph-IOP injury (Fig. 1e).

Moreover, we investigated the upstream mechanism leading to decreased FTH1 levels in ph-IOP injured mice. Ferritinophagy, a ferritin degradation process mediated by NCOA4 (the ferritin autophagic cargo-receptor), plays a vital role in controlling cellular iron homeostasis [37, 38]. To clarify the role of NCOA4 in ph-IOP-injured mice, we examined the level of endogenous NCOA4 in the retina. The observed decrease in the NCOA4 level was consistent with the reduction of FTH1 (Fig. 2e–g and Supplementary Table 2). Co-IP results showed that the endogenous interaction between NCOA4 and FTH1 was enhanced after ph-IOP injury, indicating ferritinophagy activation (Fig. 2i, j). To further demonstrate that ferritinophagy promoted FTH1 degradation and disturbed normal iron homeostasis, we used three short hairpin RNA (shRNA) to knock down NCOA4. The results showed that sh*Ncoa4*-3 had the best knockdown efficiency (87.4%; Fig. 2k, l and Supplementary Table 1). Knockdown of *Ncoa4* increased the retinal FTH1 level (Fig. 2m–o and Supplementary Table 3), accompanied by a decreased ferrous iron content in the retina after ph-IOP injury (Fig. 2p and Supplementary Table 3). These data suggested that FTH1 degradation was the leading cause of retinal intracellular iron accumulation after ph-IOP injury. NCOA4, positioned upstream of FTH1, was likely to mediate FTH1 degradation *via* ferritinophagy.

### Ph-IOP induced iron accumulation led to retinal ferroptosis at the early phase

**Fig. 3:**
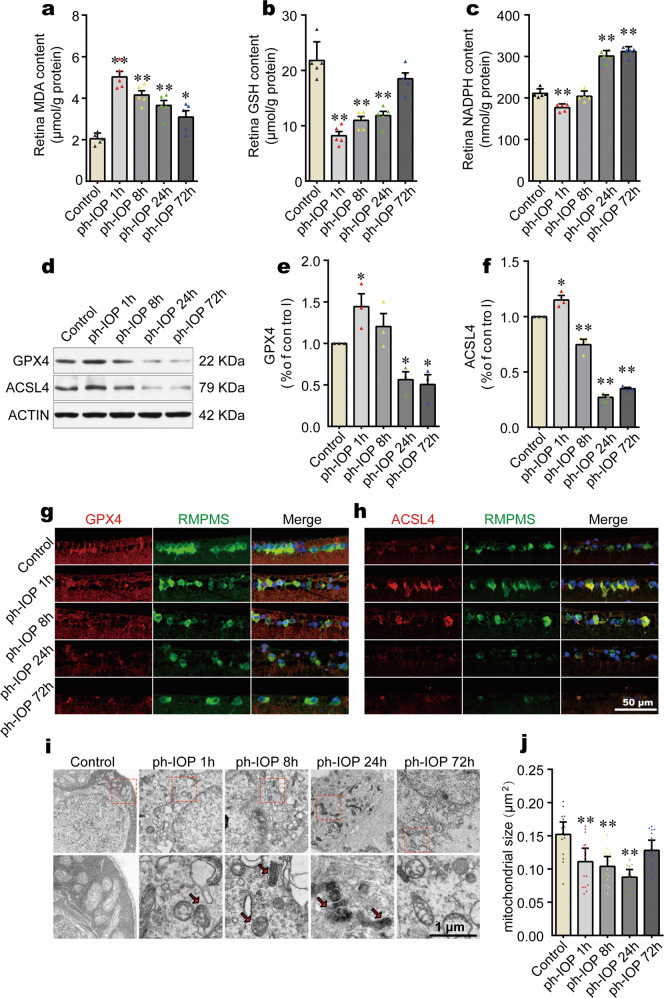
Pathologically high intraocular pressure (ph-IOP)-induced iron accumulation led to retinal ferroptosis at the early phase. Comparison of MDA (**a**), GSH (**b**), and NADPH (**c**) contents between control mice and ph-IOP injured mice (*n* = 5 in each group). **d**–**f** Western blotting detection of retinal levels of GPX4 and ACSL4 (normalized to that of actin) in control and ph-IOP injured mice (*n* = 3 in each group). Representative photomicrographs of immunofluorescence staining for GPX4 (**g**; red) and ACSL4 (**h**; red) in retinal ganglion cells (counterstained with RBPMS; green). **i** Representative photomicrographs of transmission electron microscopy showing the mitochondria with ferroptosis features (red arrows) after ph-IOP injury. **j** Comparison of mitochondrial size between control mice and ph-IOP injured mice (*n* = 15 in control, 1 h and 72 h groups; *n* = 14 in 8 h and 24 h groups). MDA malondialdehyde, GSH, glutathione, NADPH nicotinamide adenine dinucleotide phosphate, GPX4 glutathione peroxidase 4, ACSL4 Acyl-CoA synthetase long-chain family member 4, RBPMS RNA-binding protein with multiple splicing. Data are shown as the mean ± SD; *p < 0.05, **p < 0.01 (compared with the control group using one-way analysis of variance). Bar = 50 μm (**g** and **h**) and 1 μm (**i**).

Previous studies have demonstrated that elevated ferrous iron, as a strong oxidant, plays a crucial role in ferroptosis [19, 39, 40]. Thus, we hypothesized that retinal accumulated ferrous iron caused by ph-IOP could lead to retinal ferroptosis. To test this hypothesis, we determined retinal ferroptosis by measuring the contents of oxidative stress-related biochemical indicators (MDA, GSH, and NADPH [30–32]). Ph-IOP increased the MDA content significantly, and decreased the GSH and NADPH levels in the early phase after modeling (Fig. 3a–c and Supplementary Table 2). We next assessed retinal ferroptosis by evaluating core ferroptosis-related biomarkers (GPX4 and ACSL4) [41, 42]. The level of GPX4 (an endogenous antioxidant regarded as a central regulator of ferroptosis) decreased at 24 h after ph-IOP injury (Fig. 3d, e and Supplementary Table 2). Meanwhile, the level of ACSL4 (a common positive regulator of ferroptosis) was upregulated at the first hour after ph-IOP injury (Fig. 3d, f and Supplementary Table 2). Consistent with ph-IOP-induced retinal injury, the changes to these ferroptosis-related proteins were mainly concentrated in the inner layer of the retina, especially the ganglion cell layer (Fig. 3g, h and Supplemental Fig. S1), suggesting that ferroptosis-induced cell injury was likely to be concentrated in the RGCs (located in the innermost layer of the retina). We used TEM to detect whether RGCs underwent ferroptosis in the ganglion cell layer, which showed that ph-IOP caused RGC mitochondrial shrinkage and increased mitochondrial membrane density (Fig. 3i, j and Supplementary Table 2). However, these morphological features of ferroptosis were only prominent at 1, 8, and 24 h post ph-IOP injury (red arrows in Fig. 3i) and were not evident at 72 h or in control eyes.

Together, these data suggested that ph-IOP-induced retinal ferrous iron accumulation leads to retinal ferroptosis, especially in RGCs.

### Deferiprone reduced ph-IOP-induced iron accumulation

**Fig. 4:**
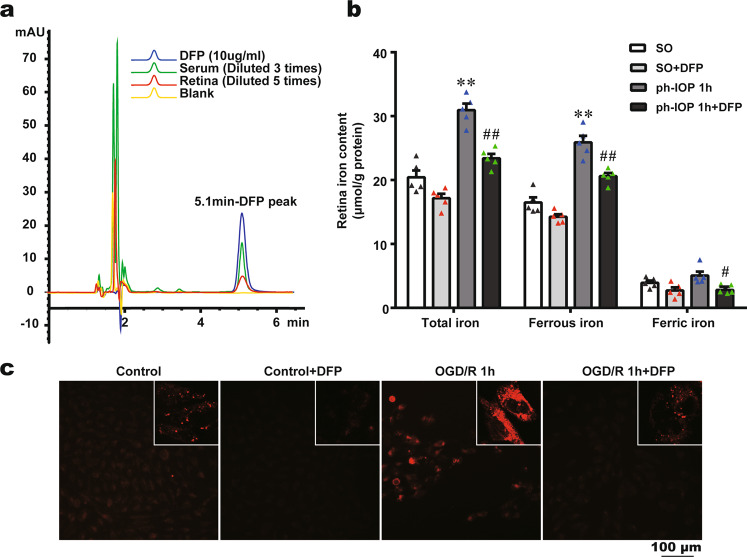
Deferiprone (DFP) treatment reduced pathologically high intraocular pressure (ph-IOP) induced iron accumulation in vitro and in vivo. **a** High-Performance Liquid Chromatography showed a detectable DFP absorption peak (at 5.1 min) both in the serum and the retina after mice were administered orally with 200 mg/kg DFP for 60 min. **b** Retinal iron content of sham operation (SO) mice and ph-IOP injured mice, with or without DFP treatment (*n* = 5 in each group). **c** Cytoplasmic ferrous iron stained using FerroOrange in the control and oxygen-glucose deprivation/ reoxygenation (OGD/R) groups, with or without DFP treatment. Data are shown as the mean ± SD; **p < 0.01 (compared with the SO group using one-way analysis of variance); #*p* < 0.05, ##*p* < 0.01 (compared with ph-IOP 1 h group using one-way analysis of variance). Bar = 100 μm.

Deferiprone (DFP; 1,2-dimethyl-3-hydroxy-4-pyridone) is a highly efficient oral iron chelator used clinically to treat iron overload diseases [43]. DFP has the lowest molecular weight among available iron-chelating agents, and its cyclic structure endows it with more efficient absorption and tissue permeability [44]. Although previous studies described the oral administration of DFP in retinal degeneration diseases [45, 46], there is no direct evidence that DFP could pass through the blood-retinal barrier. We used HPLC to verify the rationality of oral administration of DFP to treat retinal diseases. The DFP concentrations in serum and the retina after 60 min of oral administration of 200 mg/kg DFP were 39.21 ± 9.67 µg/ml and 6.19 ± 1.31 µg/g, respectively (Fig. 4a), indicating that orally-administered DFP could pass through the blood-retinal barrier.

We then examined the effect of DFP on iron metabolism in ph-IOP-injured mice. DFP treatment reduced ph-IOP-induced total iron and ferrous iron accumulation in retinas significantly (Fig. 4b and Supplementary Table 4). Moreover, similar to the in vivo results, 1 mM DFP pretreatment markedly reduced the ferrous iron content in the cytoplasm of R28 cells injured by OGD/R (Fig. 4c). These results indicated that DFP was an excellent iron chelator to treat retinal diseases; oral application of DFP could reduce ph-IOP-induced iron accumulation in vivo and in vitro.

### Chelating iron using DFP ameliorated ph-IOP induced retinal ferroptosis

**Fig. 5:**
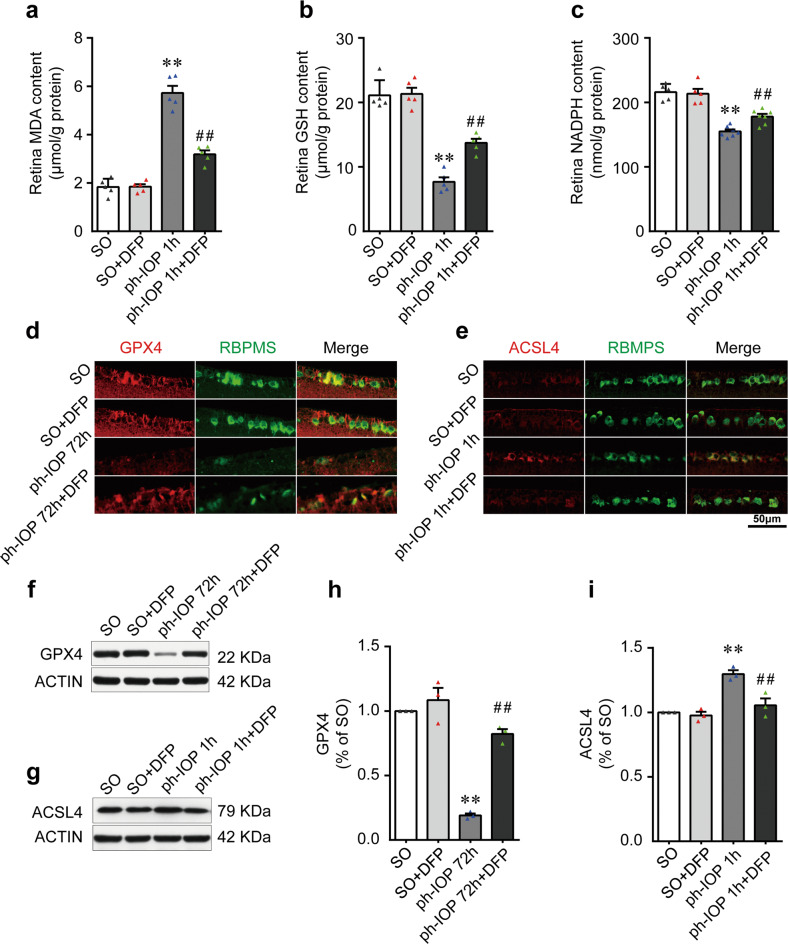
Chelating iron using deferiprone (DFP) ameliorated pathologically high intraocular pressure (ph-IOP)-induced retinal ferroptosis. Comparison of MDA (**a**), GSH (**b**), and NADPH (**c**) contents between sham operation (SO) and ph-IOP injured mice, with or without DFP treatment (*n* = 5 in each group). **d**, **e** Representative photomicrographs of immunofluorescence staining for GPX4 (**d**; red) and ACSL4 (**e**; red) in retinal ganglion cells (counterstained with RBPMS; green). **f**–**i** Western blotting detection of retinal levels of GPX4 and ACSL4 (normalized to that of actin) (*n* = 3 in each group). MDA malondialdehyde, GSH glutathione, NADPH nicotinamide adenine dinucleotide phosphate, GPX4 glutathione peroxidase 4, ACSL4 Acyl-CoA synthetase long-chain family member 4; RBPMS, RNA-binding protein with multiple splicing. Data are shown as the mean ± SD; **p < 0.01 (compared with the SO group using one-way analysis of variance); ##*p* < 0.01 (compared with the ph-IOP group using one-way analysis of variance). Bar = 50 μm.

Iron is a crucial regulator and contributor to ferroptosis, and reducing free iron using chelators is an effective method to prevent ferroptosis [20]. Thus, we assessed ferroptosis-related biomarkers in ph-IOP injured mice after DFP treatment. DFP treatment decreased retinal MDA contents (Fig. 5a and Supplementary Table 4) and increased GSH and NADPH levels after ph-IOP injury (Fig. 5b, c and Supplementary Table 4). Moreover, DFP treatment restored GPX4 levels and decreased ACSL4 levels in ph-IOP injured mice (Fig. 5d–i and Supplementary Table 4). Therefore, DFP could ameliorate ph-IOP-induced retinal ferroptosis.

### Inhibiting ferroptosis using DFP protected RGCs from ph-IOP injury

**Fig. 6:**
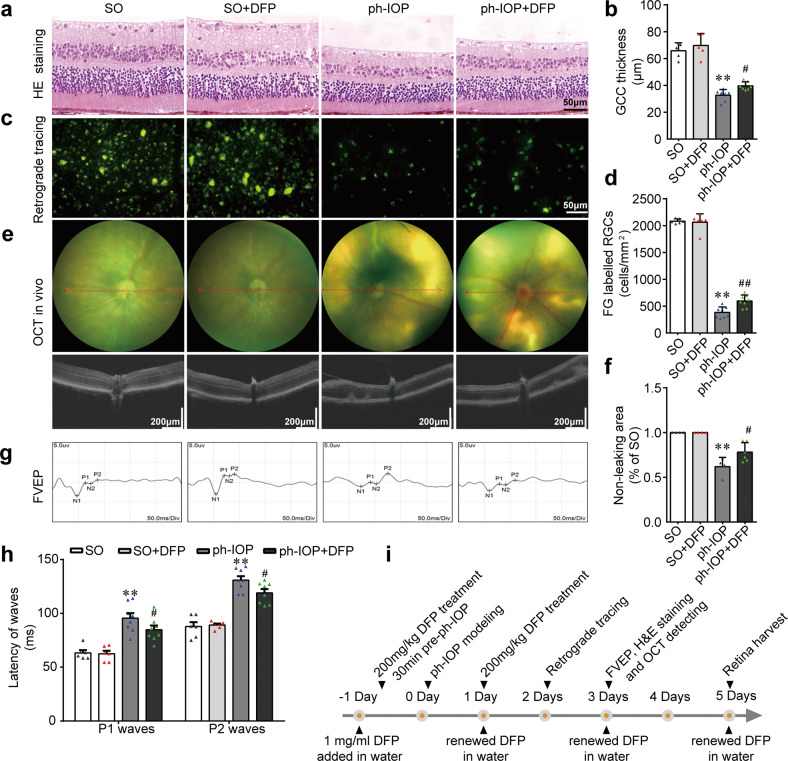
Inhibiting ferroptosis using deferiprone (DFP) protected retinal ganglion cells (RGCs) from pathologically high intraocular pressure (ph-IOP) injury. Representative photomicrographs of hematoxylin and eosin (HE)-stained retinal slices (**a**) and the thickness of the ganglion cell complex (GCC; **b**) in the sham operation (SO) mice and ph-IOP injured mice, with or without DFP treatment (*n* = 5 in SO groups and *n* = 7 in ph-IOP groups). Representative photomicrographs of fluorogold (FG)-labeled RGCs from the peripheral retinas (**c**) and the number of FG-labeled RGCs (**d**) (*n* = 6 in SO groups and *n* = 8 in ph-IOP groups). Representative photomicrographs of optical coherence tomography (OCT)-detected retinas (**e**) and non-leaking areas (**f**) (*n* = 4 in SO groups and *n* = 6 in ph-IOP groups). Representative photomicrographs of flash visual-evoked potentials (FVEPs; **g**) and the latency of P1 and P2 waves (**h**) (*n* = 6 in SO groups and *n* = 8 in ph-IOP groups). **i** Schematic diagram of the verification of the protective effect of DFP on ph-IOP-induced RGC damage. Data are shown as the mean ± SD; **p < 0.01 (compared with the SO group using one-way analysis of variance); #*p* < 0.05, ##*p* < 0.05 (compared with the ph-IOP group using one-way analysis of variance). Bar = 50 μm (**a** and **c**), 200 μm (**e**) and 50 ms (**g**).

Ph-IOP led to significant RGC loss and visual function impairment after modeling (Fig. 6a–h), and this damage became more severe over time (Supplemental Fig. S1). Although we demonstrated that ph-IOP-induced retinal iron accumulation led to retinal ferroptosis, which was ameliorated by DFP, whether DFP-induced ferroptosis inhibition could arrest RGC death required clarification. We verified the pharmaceutical effect of DFP on RGCs morphologically and functionally (Fig. 6i). DFP treatment attenuated GCC thinning (Fig. 6a, b) and increased the number of FG-labeled surviving RGCs at 5 d after ph-IOP injury (Fig. 6c, d). DFP treatment also reduced the OCT-detected retinal leakage area (Fig. 6e, f) and decreased the P1 and P2 waves’ latency after ph-IOP injury (Fig. 6g, h). These results demonstrated that DFP protected against RGC injury after ph-IOP insult (Supplementary Table 4); therefore, inhibiting ferroptosis using DFP represents an effective therapeutic strategy to treat ph-IOP-induced RGCs loss.

## Discussion

Investigating the mechanisms underlying glaucoma pathogenesis have long been a research focus in ophthalmology. Ph-IOP is widely recognized as the main pathogenic factor underlying glaucoma injuries [4]. During APACG, the obstruction of aqueous humor outflow caused by angle-closure elevated the IOP acutely in patients, directly exerting mechanical pressure on the RGCs and optic nerve [5, 23]. This results in deformation of the posterior fovea of the lamina cribrosa, compressing the axons of RGCs that pass through, which blocks the axoplasmic transport of RGCs, resulting in RGC cell death [5]. In addition, oxidative stress and glutamate excitotoxicity are also involved in the damage to RGCs under ph-IOP conditions [47, 48]. Despite the pivotal role of ph-IOP in glaucoma, control of ph-IOP alone does not entirely prevent the loss of glaucomatous RGCs, which is problematic for clinical treatment [9]. It is essential to explore the pathological mechanism responsible for RGCs loss after ph-IOP control and provide insights to develop therapeutic drugs to treat glaucoma clinically.

In our study, we simulated the injury process of ph-IOP during the onset of glaucoma by perfusing the mouse anterior chamber to mimic IOP elevation and returned the pressure to a normal level at the end of the designated time period. We observed the pathophysiological changes in the retina and RGCs after IOP normalization. The results showed that loss of RGCs continued after the ph-IOP returned to normal, which is consistent with the progression of glaucoma after IOP control in patients with glaucoma. Further studies revealed that abnormal retinal iron metabolism (increased free ferrous iron) is a critical reason for RGC loss after ph-IOP recovery. Physiologically, iron in the retina is always maintained at a relatively stable level [13]. Excessive iron could cause cytotoxicity, while iron deficiency could cause cellular dysfunction [49]. Iron metabolism regulation mainly involves iron uptake, iron excretion, and iron storage. Thus, to identify the possible determinants of abnormal iron metabolism after ph-IOP injury, we must consider these three factors comprehensively.

Retinal cellular iron uptake is dependent on TR on the cell membrane. TR recognizes transferrin (TF) in the systemic circulation and helps internalize TF into the cell *via* endocytosis [50]. Subsequently, TF dissociates and releases iron atoms, completing the transport of iron from the extracellular to the intracellular space. In addition to TR, DMT1 has also been implicated in cellular iron uptake. DMT1 can assist endocytosis to release cytosolic iron for cellular uptake, and DMT1 dysfunction can lead to low tissue iron uptake, while its overexpression leads to excessive iron uptake [51, 52]. Our investigations showed downregulation of both TR and DMT1 within the retina after ph-IOP injury, suggesting suppression the inner retinal iron uptake capacity. This contradicts the observation of increased internal retinal iron content after ph-IOP injury, indicating that the iron uptake pathway was not responsible for the abnormal retinal iron metabolism.

Iron excretion from retinal cells mainly depends on Fpn1, which is expressed widely in the retinal ganglion cell layer, the inner and outer plexiform layer, the photoreceptor cell layer, and the pigment epithelium layer [53]. Currently, Fpn1 is the only known iron excretion channel. In our study, the Fpn1 content was downregulated significantly at 8–72 h after ph-IOP injury, suggesting that inhibition of the iron excretion pathway might be one of the reasons behind the accumulation of retinal iron. However, this result could not explain the pathological phenomenon that ferrous iron in the retina reached a peak concentration at 1 h after ph-IOP injury.

Ferritin degradation is the main reason for the accumulation of ferrous iron after ph-IOP injury. Ferritin is the most critical iron storage protein in cells. It is divided into heavy ferritin chains (Ferritin heavy chains, FTH1) and ferritin light chains (Ferritin light chains, FLH) [54]. One ferritin molecule can bind 4500 iron atoms; thus it is also termed the intracellular iron pool [13]. Our study showed that the of FTH1 level downregulated significantly at 1 h after ph-IOP injury, indicating that iron originally bound to ferritin was released into the cytoplasm, resulting in a sharp increase in the retinal free ferrous iron content. Previous studies showed that autophagy, specifically NCOA4-mediated autophagy (terminated ferritinophagy), plays a vital role in recognizing ferritin and delivering it for lysosomal degradation with the help of NCOA4 [37, 38, 55]. However, the role of NCOA4 and ferritinophagy in glaucoma is unknown. Additionally, our results showed that NCOA4 and FTH1 were both degraded after ph-IOP injury. In vivo knockdown of *Ncoa4* increased retinal FTH1 levels and reduced free iron levels. These results suggested that NCOA4-mediated ferritinophagy is the upstream mechanism of FTH1 degradation and abnormal iron metabolism after ph-IOP injury.

Notably, ischemia and hypoxia injury is one of the mechanisms of ph-IOP injury [5]. After ph-IOP control, retinal blood perfusion increases, which will inevitably lead to RGC ischemia-reperfusion injury [23]. In fact, ischemia-reperfusion injury and iron metabolism are also closely correlated. Studies show that ischemia-reperfusion injury of the brain, intestine, and kidney is also accompanied by abnormal iron metabolism [56–58], consistent with the conclusion of this study. Unlike previous studies, we further refined which type of iron ions resulted in iron accumulation. Interestingly, the elevated free iron in the retina is mainly ferrous iron; however, that in serum is primarily ferric iron. One possible explanation is that the free iron in cells cannot be directly discharged out of cells in the form of ferric iron, but relies on ceruloplasmin and hephaestin to catalyze ferrous iron into ferric iron [59, 60]. This could also explain why the time of free iron elevation in serum lags behind that in the retina.

Ferroptosis is an iron-dependent regulatory necrotic process [19]. Disordered iron metabolism (especially increased ferrous iron) is the initiating factor of ferroptosis. Free ferrous iron is a solid oxidative factor that produces hydroxyl radicals through the Fenton reaction [13]. These unstable hydroxyl radicals can oxidize lipid metabolites, such as polyunsaturated fatty acids, into cytotoxic lipid peroxides, thus promoting ferroptosis [39, 40]. Iron can also collaborate with ROS-producing enzymes, such as NADPH oxidases (NOXs), xanthine oxidases, lipoxygenases (LOXs), and cytochrome P450 enzymes to promote the production of intracellular ROS [61]. Elevated ferrous iron will eventually increase intracellular lipid peroxides (as indicated by MDA levels). Ferroptosis occurs when lethal lipid peroxides exceed the upper limit of cellular clearance (such as the GSH reduction system involved in GPX4) [20, 21]. In our study, we found that with the increase in retinal ferrous iron, the MDA content increased and the GSH content decreased. After chelating ferrous iron with DFP, the MDA and GSH contents were restored to a great extent. These results indicated that retinal ferrous iron accumulation is the upstream initiating factor of ferroptosis after ph-IOP injury.

The blood-retinal barrier is a unique barrier that plays a significant role in maintaining the homeostasis of the retinal microenvironment [62]. However, despite shielding the retina from harmful substances, it also hinders the entry of a majority of beneficial drugs into the retina. To block retinal elevated ferrous iron after ph-IOP injury, we required an iron-chelating drug that can pass through the blood-retinal barrier. Presently, three kinds of iron chelators are commonly used in the clinic: DFP, deferoxamine, and deferasirox. Among them, DFP has the smallest molecular weight [44]. Its circular molecular structure allows for better tissue compatibility and cell trafficability, making it the most hopeful candidate to pass through the blood-retinal barrier. The HPLC results showed significant DFP residues in the retina of mice after oral administration, which confirmed our speculation. In addition, DFP can be administered orally, effectively avoiding a series of complications caused by intravitreal injection, and making it advantageous for subsequent clinical treatment [63].

Although DFP inhibited ferroptosis and reduced RGC loss caused by ph-IOP, its therapeutic effect was relatively limited. In addition to ferroptosis first explored in this study, glaucoma causes death *via* apoptosis, necroptosis, pyroptosis, and other types of cell death [64–66]; therefore, no single drug can yet treat all kinds of cell death modes. Furthermore, different cell death types have differ occurrence time-spans and progression patterns [67], ultimately leading to difficulty in achieving satisfactory results in the protective treatment of RGCs. In essence, clarifying the functional characteristics of ferroptosis in the loss of RGCs after ph-IOP will help us better understand the pathogenesis of glaucoma and allow the formulation of comprehensive glaucoma treatment strategies in the future.

Our study found that ferroptosis is mainly involved in the early stage after ph-IOP injury. The ferroptosis inducer, ferrous iron, primarily came from FTH1-bound iron in the retina; therefore, the amount of stored iron was limited. With the continuous degradation of FTH1, the release of stored iron gradually decreased and was eventually stabilized. However, this transient elevation in ferrous iron disrupts the redox system in the cytoplasm through the Fenton reaction, leading to the accumulation of lethal lipid peroxides, and eventually resulting in RGC ferroptosis. Among all the death modes of RGCs explored, apoptosis is the most studied and is considered the main form of death of RGCs [68]. However, apoptosis involves a complex cascade of regulatory pathways from extracellular, to cytoplasmic, to nuclear, such as the binding of death receptors and ligands, the transcription and translation of apoptosis-related genes, the assembly of death domain proteins, and the activation of caspase effectors, which together make RGCs apoptosis relatively lagging and persistent [69, 70]. In addition, intracellular metabolic stress is a fundamental reason for activating the intrinsic apoptosis pathway [71]. The imbalance of redox metabolism related to ferroptosis might also be involved in the occurrence and development of RGC apoptosis; however, the relationship between them requires further examination.

**Fig. 7:**
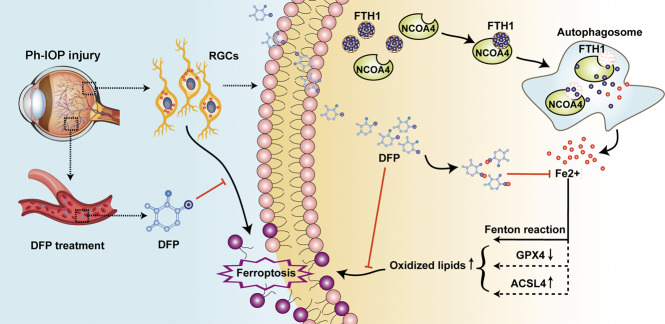
The putative signaling pathways for deferiprone (DFP) in inhibiting pathologically high intraocular pressure (ph-IOP) induced retinal ganglion cell (RGC) ferroptosis by chelating the free iron released by Nuclear receptor coactivator 4 (NCOA4)-mediated FTH1 (ferritin heavy polypeptide 1) degradation. In the physiological situation, intracellular iron is in dynamic balance; however, this balance will be broken in the pathological state. Ph-IOP injury can activate intracellular NCOA4 in RGCs, and then activated NCOA4 will combine the FTH1 (the main intracellular iron storage protein) and trigger FTH1 degradation by lysosomes (known as ferritinophagy). Degraded FTH1 will release large amounts of free iron; this released iron is a redox-active metal and is further involved in lipid peroxidation. Ultimately, the accumulation of lipid hydroperoxides caused by iron leads to RGC ferroptosis. However, oral administration of DFP can chelate superfluous intracellular free iron and inhibit ph-IOP-induced RGC ferroptosis.

In conclusion, our study revealed the function and features of ferroptosis in glaucoma (Fig. 7). We found that Ph-IOP could disturb iron homeostasis, leading to excessive ferrous iron accumulation in the retina at the early phase after injury. This ferrous iron accumulation disrupts the intracellular redox system, triggering RGC ferroptosis. NCOA4-mediated FTH1 degradation is one explanation for the intracellular ferrous iron accumulation after ph-IOP, because knockdown of *Ncoa4* inhibited FTH1 degradation, thereby alleviating ferrous iron accumulation. Orally administrated DFP could penetrate the blood-retinal barrier to effectively chelate excess retinal ferrous iron, ultimately inhibiting RGC ferroptosis. Moreover, DFP treatment had a delectable protective effect on RGC morphology and function. These findings suggest that ferroptosis is a new target for glaucoma treatment, especially in treating continued loss of RGCs in glaucoma after ph-IOP control.

## Supplementary information

Detailed Author Contribution form

Reproducibility Checklist

Supplemental Table 1

Supplemental Table 2

Supplemental Table 3

Supplemental Table 4

Supplemental Figure 1

Supplemental Figure 2

Supplemental Figure 3

Supplemental Figure legends

Supplementary File 1

## Data Availability

RNA-seq data in this study have been deposited in Sequence Read Archive of NCBI with the accession code PRJNA838649. All other datasets generated and/or analyzed during the current study are available at https://figshare.com/s/c635f9585c43f7992591.
