# Supplementary material for: Pathologically high intraocular pressure disturbs normal iron homeostasis and leads to retinal ganglion cell ferroptosis in glaucoma
Source: Cell Death Differ. 2022 Aug 6;30(1):69–81. doi: 10.1038/s41418-022-01046-4 (PMC9883496; doi:10.1038/s41418-022-01046-4)
Supplement: Supplementary file 1 — Detailed Author Contribution form [file 41418_2022_1046_MOESM1_ESM.pdf]

DECLARATION OF CONTRIBUTIONS TO ARTICLE

ADMC

Manuscript Number: CDD-21-2546R

Journal Name: *Cell Death & Differentiation* (the 'Journal')

Proposed Title of the Contribution:

Pathologically high intraocular pressure disturbs normal iron homeostasis and leads to retinal ganglion cell ferroptosis in glaucoma (the 'Contribution)

Author(s):

Fei Yao, Jingjie Peng, Endong Zhang, Dan Ji, Zhaolin Gao, Yixiong Tang, Xueyan Yao, Xiaobo Xia (the 'Authors')

For all *CDD* articles, each person named as an author in the published version must be able to show he or she has contributed substantially to the article.

Authorship credit should be based on 1) substantial contributions to conception and design, acquisition of data, or analysis and interpretation of data; 2) drafting the article or revising it critically for important intellectual content; and 3) final approval of the version to be published. Authors should meet conditions 1, 2 and 3.

Any person who cannot be shown to have made a substantial contribution to the article cannot be listed as an author in the final version. The name of any person who is deemed to have made a minor contribution can, however, appear in the Acknowledgments section of the article.

Please complete the table below to indicate the contributions of all named authors to the manuscript.

Author Full Name:	Specification of Contribution to the Manuscript:
Fei Yao	Conceived and designed the study; Developed the methodology and performed the writing; Acquired the data.
Jingjie Peng	Developed the methodology and performed the writing, review, and revision of the paper
Endong Zhang	Conceived and designed the study;Developed the methodology.
Dan Ji	Conceived and designed the study;Analyzed and interpreted the data.
Zhaolin Gao	Developed the methodology and performed the revision of the paper.
Yixiong Tang	Developed the methodology and performed the revision of the paper.
Xueyan Yao	Developed the methodology and performed the revision of the paper.
Xiaobo Xia	Conceived and designed the study; Performed the statistical analysis; Provided technical and material support.

Please complete the table below to indicate the contributions of all named authors to the figures.

Figure 1:

Fei Yao, Dan Ji, Zhaolin Gao, Yixiong Tang, Xiaobo Xia

Figure 2:

Fei Yao, Jingjie Peng, Endong Zhang, Dan Ji, Xiaobo Xia

Figure 3:

Fei Yao, Jingjie Peng, Endong Zhang, Dan Ji, Xiaobo Xia

Figure 4:

Fei Yao, Endong Zhang, Dan Ji, Xiaobo Xia

Figure 5:

Fei Yao, Jingjie Peng, Endong Zhang, Dan Ji, Xiaobo Xia

Figure 6:

Fei Yao, Endong Zhang, Dan Ji, Zhaolin Gao, Yixiong Tang, Xueyan Yao, Xiaobo Xia

Signed for and on behalf of the Author(s):	Print Name:	Date:
Xiaobo Xia	Xiaobo Xia	2022.5.30
