# Supplementary material for: Pathologically high intraocular pressure disturbs normal iron homeostasis and leads to retinal ganglion cell ferroptosis in glaucoma
Source: Cell Death Differ. 2022 Aug 6;30(1):69–81. doi: 10.1038/s41418-022-01046-4 (PMC9883496; doi:10.1038/s41418-022-01046-4)
Supplement: Supplementary file 3 — Supplemental Table 1 [file 41418_2022_1046_MOESM3_ESM.docx]

**Supplemental Table 1. Changes in retinal NCOA4 levels in normal mice receiving AAV-shRNA injection.**

| **Retinal NCOA4 levels**  **(% of Control)** | **Groups** | | | |
| --- | --- | --- | --- | --- |
|  | **Control** | **sh*Ncoa4*-1** | **sh*Ncoa4*-2** | **sh*Ncoa4*-3** |
| Values | 1.00 ± 0.00 | 0.27 ± 0.04** | 0.49 ± 0.03** | 0.13 ± 0.01** |
| Sample sizes | 3 | 3 | 3 | 3 |
| *P* values | / | 0.000 *vs* Control | 0.000 *vs* Control | 0.000 *vs* Control |

Data are the mean ± SD unless stated otherwise.

AAV, Adeno-associated virus; NCOA4, nuclear receptor coactivator 4; shRNA, short hairpin ribonucleic acid.

AAV vector sequences:

Control: 5′-ACCGCCTAAGGTTAAGTCGCCCTCGCTCGAGCGAGGGCGACTTAACCTTAGGTTTTTTG-3′;

sh*Ncoa4*-1: 5′-ACCGGGAAAGGACAAGAATGGAATTCAAGAGATTCCATTCTTGTCCTTTCCTTTTTTG-3′;

sh*Ncoa4*-2: 5′-ACCGCCAGAGCAGAAGTCAGCATTTCAAGAGAATGCTGACTTCTGCTCTGGTTTTTTG-3′;

sh*Ncoa4*-3: 5′-ACCGCCTTGTCAGAGTGGCTTATTTCAAGAGAATAAGCCACTCTGACAAGGTTTTTTG-3′.

***p* < 0.01 (sh*Ncoa4* group compared with control group using one-way analysis of variance).
