# Supplementary material for: Pathologically high intraocular pressure disturbs normal iron homeostasis and leads to retinal ganglion cell ferroptosis in glaucoma
Source: Cell Death Differ. 2022 Aug 6;30(1):69–81. doi: 10.1038/s41418-022-01046-4 (PMC9883496; doi:10.1038/s41418-022-01046-4)
Supplement: Supplementary file 4 — Supplemental Table 2 [file 41418_2022_1046_MOESM4_ESM.docx]

**Supplemental Table 2. Changes in parameters in control mice and** **pathologically high intraocular pressure (ph-IOP) injured mice at 1, 8, 24, and 72 hours after modeling.**

| **Parameters** | | **Groups** | | | | |
| --- | --- | --- | --- | --- | --- | --- |
|  |  | **Control** | **ph-IOP 1h** | **ph-IOP 8h** | **ph-IOP 24h** | **ph-IOP 72h** |
| **Retinal total iron contents**  **(μmol/g protein)** | Values | 21.11 ± 2.81 | 31.62 ± 3.29** | 26.33 ± 1.20* | 20.94 ± 1.47 | 23.40 ± 1.58 |
|  | Sample sizes | 5 | 5 | 5 | 5 | 5 |
|  | *P* values | / | 0.000 *vs* control | 0.011 *vs* control | 1.000 *vs* control | 0.498 *vs* control |
| **Retinal ferrous iron contents**  **(μmol/g protein)** | Values | 17.46 ± 2.05 | 26.31 ± 1.44** | 22.12 ± 0.75** | 18.51 ± 2.38 | 17.64 ± 1.71 |
|  | Sample sizes | 5 | 5 | 5 | 5 | 5 |
|  | *P* values | / | 0.000 *vs* control | 0.004 *vs* control | 0.878 *vs* control | 1.000 *vs* control |
| **Retinal ferric iron contents**  **(μmol/g protein)** | Values | 3.65 ± 0.88 | 5.31 ± 3.69 | 4.21 ± 0.70 | 2.44 ± 1.47 | 5.77 ± 2.99 |
|  | Sample sizes | 5 | 5 | 5 | 5 | 5 |
|  | *P* values | / | 0.776 *vs* control | 0.995 *vs* control | 0.915 *vs* control | 0.592 *vs* control |
| **Serum total iron contents**  **(nmol/ml)** | Values | 61.40 ± 4.80 | 73.48 ± 5.24 | 71.36 ± 10.61 | 66.63 ± 7.76 | 54.27 ± 8.70 |
|  | Sample sizes | 5 | 5 | 5 | 5 | 5 |
|  | *P* values | / | 0.138 *vs* control | 0.285 *vs* control | 0.819 *vs* control | 0.600 *vs* control |
| **Serum ferrous iron contents**  **(nmol/ml)** | Values | 57.41 ± 5.62 | 64.25 ± 4.84 | 54.05 ± 2.05 | 57.40 ± 5.68 | 47.56 ± 6.77 |
|  | Sample sizes | 5 | 5 | 5 | 5 | 5 |
|  | *P* values | / | 0.275 *vs* control | 0.846 *vs* control | 1.000 *vs* control | 0.052 *vs* control |
| **Serum ferric iron contents**  **(nmol/ml)** | Values | 3.98 ± 1.88 | 9.23 ± 4.23 | 17.31 ± 9.05** | 9.23 ± 5.05 | 6.72 ± 2.68 |
|  | Sample sizes | 5 | 5 | 5 | 5 | 5 |
|  | *P* values | / | 0.520 *vs* control | 0.005 *vs* control | 0.520 *vs* control | 0.919 *vs* control |
| **MDA contents**  **(μmol/g protein)** | Values | 2.05 ± 0.27 | 5.03 ± 0.59** | 4.16 ± 0.46** | 3.65 ± 0.53** | 3.10 ± 0.69* |
|  | Sample sizes | 5 | 5 | 5 | 5 | 5 |
|  | *P* values | / | 0.000 *vs* control | 0.000 *vs* control | 0.001 *vs* control | 0.036 *vs* control |
| **GSH contents**  **(μmol/g protein)** | Values | 21.79 ± 3.37 | 8.21 ± 1.67** | 10.97 ± 1.58** | 11.85 ± 1.70** | 18.50 ± 2.34 |
|  | Sample sizes | 5 | 5 | 5 | 5 | 5 |
|  | *P* values | / | 0.000 *vs* control | 0.000 *vs* control | 0.000 *vs* control | 0.178 *vs* control |
| **NADPH contents**  **(nmol/g protein)** | Values | 210.8 ± 11.2 | 176.7 ± 9.1** | 204.1 ± 12.4 | 300.8 ± 13.3** | 311.8 ± 11.7** |
|  | Sample sizes | 5 | 5 | 5 | 5 | 5 |
|  | *P* values | / | 0.001 *vs* control | 0.890 *vs* control | 0.000 *vs* control | 0.000 *vs* control |
| **Mitochondrial size (μm^2^)** | Values | 0.152 ± 0.034 | 0.111 ± 0.037 | 0.104 ± 0.026 | 0.088 ± 0.020 | 0.128 ± 0.028 |
|  | Sample sizes | 15 | 15 | 14 | 14 | 15 |
|  | *P* values | / | 0.003 *vs* control | 0.000 *vs* control | 0.000 *vs* control | 0.179 *vs* control |
| **GPX4 levels**  **(% of control)** | Values | 1.00 ± 0.00 | 1.44 ± 0.27* | 1.20 ± 0.27 | 0.56 ± 0.17* | 0.51 ± 0.20* |
|  | Sample sizes | 3 | 3 | 3 | 3 | 3 |
|  | *P* values | / | 0.026 *vs* control | 0.259 *vs* control | 0.028 *vs* control | 0.015 *vs* control |
| **ACSL4 levels**  **(% of control )** | Values | 1.00 ± 0.00 | 1.15 ± 0.07* | 0.75 ± 0.08** | 0.27 ± 0.04** | 0.35 ± 0.02** |
|  | Sample sizes | 3 | 3 | 3 | 3 | 3 |
|  | *P* values | / | 0.038 *vs* control | 0.001 *vs* control | 0.000 *vs* control | 0.000 *vs* control |
| **DMT1 levels**  **(% of control )** | Values | 1.00 ± 0.00 | 0.99 ± 0.05 | 0.35 ± 0.09** | 0.41 ± 0.14** | 0.33 ± 0.10** |
|  | Sample sizes | 3 | 3 | 3 | 3 | 3 |
|  | *P* values | / | 1.000 *vs* control | 0.000 *vs* control | 0.000 *vs* control | 0.000 *vs* control |
| **Fpn1 levels**  **(% of control )** | Values | 1.00 ± 0.00 | 0.76 ± 0.24 | 0.54 ± 0.18* | 0.46 ± 0.13* | 0.30 ± 0.16** |
|  | Sample sizes | 3 | 3 | 3 | 3 | 3 |
|  | *P* values | / | 0.414 *vs* control | 0.036 *vs* control | 0.014 *vs* control | 0.002 *vs* control |
| **TR levels**  **(% of control )** | Values | 1.00 ± 0.00 | 0.96 ± 0.02 | 0.83 ± 0.11* | 0.66 ± 0.05** | 0.59 ± 0.03** |
|  | Sample sizes | 3 | 3 | 3 | 3 | 3 |
|  | *P* values | / | 0.907 *vs* control | 0.022 *vs* control | 0.000 *vs* control | 0.000 *vs* control |
| **FTH1 levels**  **(% of control )** | Values | 1.00 ± 0.00 | 0.56 ± 0.05** | 0.19 ± 0.03** | 0.35 ± 0.15** | 0.26 ± 0.09** |
|  | Sample sizes | 3 | 3 | 3 | 3 | 3 |
|  | *P* values | / | 0.001 *vs* control | 0.000 *vs* control | 0.000 *vs* control | 0.000 *vs* control |
| **NCOA4 levels**  **(% of control )** | Values | 1.00 ± 0.00 | 0.65 ± 0.13* | 0.45 ± 0.12** | 0.40 ± 0.15** | 0.42 ± 0.17** |
|  | Sample sizes | 3 | 3 | 3 | 3 | 3 |
|  | *P* values | / | 0.048 *vs* control | 0.003 *vs* control | 0.001 *vs* control | 0.002 *vs* control |

Data are the mean ± SD unless stated otherwise.

ACSL4, acyl-CoA synthetase long-chain family member 4; DMT1, divalent metal transporter 1; Fpn1, ferroportin 1; FTH1, ferritin heavy polypeptide 1; GPX4, glutathione peroxidase 4; GSH, glutathione; MDA, malondialdehyde; NADPH, nicotinamide adenine dinucleotide phosphate; NCOA4, nuclear receptor coactivator 4; TR, transferrin receptor.

**p* < 0.05, ***p* < 0.01 (ph-IOP group compared with control group using one-way analysis of variance).
