# Supplementary material for: Pathologically high intraocular pressure disturbs normal iron homeostasis and leads to retinal ganglion cell ferroptosis in glaucoma
Source: Cell Death Differ. 2022 Aug 6;30(1):69–81. doi: 10.1038/s41418-022-01046-4 (PMC9883496; doi:10.1038/s41418-022-01046-4)
Supplement: Supplementary file 5 — Supplemental Table 3 [file 41418_2022_1046_MOESM5_ESM.docx]

**Supplemental Table 3. Changes in retinal parameters in pathologically high intraocular pressure (ph-IOP) injured mice receiving AAV-shRNA injection.**

| **Retinal parameters** | | **Groups** | | |
| --- | --- | --- | --- | --- |
|  |  | **ph-IOP** | **ph-IOP + sh*Ncoa4*-3** | **ph-IOP + shControl** |
| **NCOA4 levels**  **(% of ph-IOP )** | Values | 1.00 ± 0.00 | 0.29 ± 0.04** | 0.86 ± 0.05^##^ |
|  | Sample sizes | 3 | 3 | 3 |
|  | *P* values | / | 0.000 *vs* ph-IOP | 0.000 *vs* sh*Ncoa4*-3 |
| **FTH1 levels**  **(% of ph-IOP )** | Values | 1.00 ± 0.00 | 1.89 ± 0.28** | 1.01 ± 0.20^##^ |
|  | Sample sizes | 3 | 3 | 3 |
|  | *P* values | / | 0.004 *vs* ph-IOP | 0.004 *vs* sh*Ncoa4*-3 |
| **Total iron contents**  **(μmol/g protein)** | Values | 31.57 ± 3.02 | 23.29 ± 3.81* | 33.59 ± 4.74^##^ |
|  | Sample sizes | 5 | 5 | 5 |
|  | *P* values | / | 0.015 *vs* ph-IOP | 0.004 *vs* sh*Ncoa4*-3 |
| **Ferrous iron contents**  **(μmol/g protein)** | Values | 26.93 ± 3.11 | 19.57 ± 2.35* | 29.8 ± 5.46^##^ |
|  | Sample sizes | 5 | 5 | 5 |
|  | *P* values | / | 0.028 *vs* ph-IOP | 0.003 *vs* sh*Ncoa4*-3 |
| **Ferric iron contents**  **(μmol/g protein)** | Values | 4.64 ± 1.19 | 3.71 ± 1.66 | 3.76 ± 1.08 |
|  | Sample sizes | 5 | 5 | 5 |
|  | *P* values | / | 0.536 *vs* ph-IOP | 0.998 *vs* sh*Ncoa4*-3 |

Data are the mean ± SD unless stated otherwise.

AAV, Adeno-associated virus; FTH1, ferritin heavy polypeptide 1; NCOA4, nuclear receptor coactivator 4; shRNA, short hairpin ribonucleic acid.

**p* < 0.05, ***p* < 0.01 (sh*Ncoa4-3* group compared with ph-IOP group using one-way analysis of variance); ^##^ *p* < 0.01 (shControl group compared with sh*Ncoa4*-3 group using one-way analysis of variance).
