# Supplementary material for: Pathologically high intraocular pressure disturbs normal iron homeostasis and leads to retinal ganglion cell ferroptosis in glaucoma
Source: Cell Death Differ. 2022 Aug 6;30(1):69–81. doi: 10.1038/s41418-022-01046-4 (PMC9883496; doi:10.1038/s41418-022-01046-4)
Supplement: Supplementary file 6 — Supplemental Table 4 [file 41418_2022_1046_MOESM6_ESM.docx]

**Supplemental Table 4. Changes in retinal parameters after sham operation (SO) or pathologically high intraocular pressure (ph-IOP) modeling, with or without deferiprone (DFP) treatment.**

| **Retinal parameters** | | **Groups** | | | |
| --- | --- | --- | --- | --- | --- |
|  |  | **SO** | **SO + DFP** | **ph-IOP** | **ph-IOP + DFP** |
| **Total iron contents**  **(μmol/g protein)** | Values | 20.43 ± 2.37 | 17.17 ± 1.53 | 30.94 ± 2.26** | 23.42 ± 1.51^##^ |
|  | Sample sizes | 5 | 5 | 5 | 5 |
|  | *P* values | / | 0.077 *vs* SO | 0.000 *vs* SO | 0.000 *vs* ph-IOP |
| **Ferrous iron contents**  **(μmol/g protein)** | Values | 16.51 ± 1.76 | 14.25 ± 0.94 | 25.90 ± 2.30** | 20.61 ± 1.14^##^ |
|  | Sample sizes | 5 | 5 | 5 | 5 |
|  | *P* values | / | 0.165 *vs* SO | 0.000 *vs* SO | 0.001 *vs* ph-IOP |
| **Ferric iron contents**  **(μmol/g protein)** | Values | 3.93 ± 0.77 | 2.93 ± 1.29 | 5.05 ± 1.42 | 2.81 ± 0.73^#^ |
|  | Sample sizes | 5 | 5 | 5 | 5 |
|  | *P* values | / | 0.492 *vs* SO | 0.398 *vs* SO | 0.024 *vs* ph-IOP |
| **MDA contents**  **(μmol/g protein)** | Values | 1.83 ± 0.34 | 1.85 ± 0.22 | 5.72 ± 0.66** | 3.19 ± 0.35^##^ |
|  | Sample sizes | 5 | 5 | 5 | 5 |
|  | *P* values | / | 1.000 *vs* SO | 0.000 *vs* SO | 0.000 *vs* ph-IOP |
| **GSH contents**  **(μmol/g protein)** | Values | 21.08 ± 2.36 | 21.33 ± 2.09 | 7.67 ± 1.57** | 13.72 ± 1.43^##^ |
|  | Sample sizes | 5 | 5 | 5 | 5 |
|  | *P* values | / | 0.997 *vs* SO | 0.000 *vs* SO | 0.001 *vs* ph-IOP |
| **NADPH contents**  **(nmol/g protein)** | Values | 216.02 ± 12.60 | 213.42 ± 16.59 | 154.88 ± 8.17** | 177.98 ± 11.12^##^ |
|  | Sample sizes | 5 | 5 | 7 | 7 |
|  | *P* values | / | 0.986 *vs* SO | 0.000 *vs* SO | 0.009 *vs* ph-IOP |
| **GPX4 levels**  **(% of SO)** | Values | 1.00 ± 0.00 | 1.08 ± 0.16 | 0.19 ± 0.03** | 0.82 ± 0.07^##^ |
|  | Sample sizes | 3 | 3 | 3 | 3 |
|  | *P* values | / | 0.667 *vs* SO | 0.000 *vs* SO | 0.000 *vs* ph-IOP |
| **ACSL4 levels**  **(% of SO)** | Values | 1.00 ± 0.00 | 0.97 ± 0.05 | 1.30 ± 0.05** | 1.05 ± 0.09^##^ |
|  | Sample sizes | 3 | 3 | 3 | 3 |
|  | *P* values | / | 0.959 *vs* SO | 0.001 *vs* SO | 0.004 *vs* ph-IOP |
| **FG-labeled RGCs**  **(cells/mm^2^)** | Values | 2082.8 ± 43.9 | 2063.6 ± 155.5 | 383.4 ± 101.7** | 597.0 ± 111.5^##^ |
|  | Sample sizes | 6 | 6 | 8 | 8 |
|  | *P* values | / | 0.990 *vs* SO | 0.000 *vs* SO | 0.004 *vs* ph-IOP |
| **GCC thickness**  **(μm)** | Values | 65.86 ± 5.87 | 69.77 ± 8.84 | 32.67 ± 4.23** | 39.64 ± 3.03^#^ |
|  | Sample sizes | 5 | 5 | 7 | 7 |
|  | *P* values | / | 0.277 *vs* SO | 0.000 *vs* SO | 0.029 *vs* ph-IOP |
| **Non-leaking area**  **(% of SO)** | Values | 1.00 ± 0.00 | 1.00 ± 0.00 | 0.62 ± 0.10** | 0.78 ± 0.11^#^ |
|  | Sample sizes | 4 | 4 | 6 | 6 |
|  | *P* values | / | 1.000 *vs* SO | 0.000 *vs* SO | 0.031 *vs* ph-IOP |
| **Latency of P1 waves**  **(ms)** | Values | 63.33 ± 6.19 | 62.42 ± 6.95 | 95.50 ± 13.27** | 84.81 ± 10.98^#^ |
|  | Sample sizes | 6 | 6 | 8 | 8 |
|  | *P* values | / | 0.878 *vs* SO | 0.000 *vs* SO | 0.047 *vs* ph-IOP |
| **Latency of P2 waves**  **(ms)** | Values | 87.75 ± 10.11 | 89.17 ± 3.39 | 130.88 ± 10.37** | 119.06 ± 9.76^#^ |
|  | Sample sizes | 6 | 6 | 8 | 8 |
|  | *P* values | / | 0.790 *vs* SO | 0.000 *vs* SO | 0.016 *vs* ph-IOP |

Data are the mean ± SD unless stated otherwise.

ACSL4, acyl-CoA synthetase long-chain family member 4; FG, fluorogold; GCC, ganglion cell complex; GPX4, glutathione peroxidase 4; GSH, glutathione; MDA, malondialdehyde; NADPH, nicotinamide adenine dinucleotide phosphate; P1 wave, first positive wave of flash visual-evoked potentials; P2 wave, second positive wave of flash visual-evoked potentials; RGCs, retinal ganglion cells.

***p* < 0.01 (ph-IOP group compared with SO group using one-way analysis of variance); ^#^ *p* < 0.05, ^##^ *p* < 0.01 (ph-IOP + DFP group compared with ph-IOP group using one-way analysis of variance).
