# Supplementary figures and images for: Pathologically high intraocular pressure disturbs normal iron homeostasis and leads to retinal ganglion cell ferroptosis in glaucoma
Source: Cell Death Differ. 2022 Aug 6;30(1):69–81. doi: 10.1038/s41418-022-01046-4 (PMC9883496; doi:10.1038/s41418-022-01046-4)

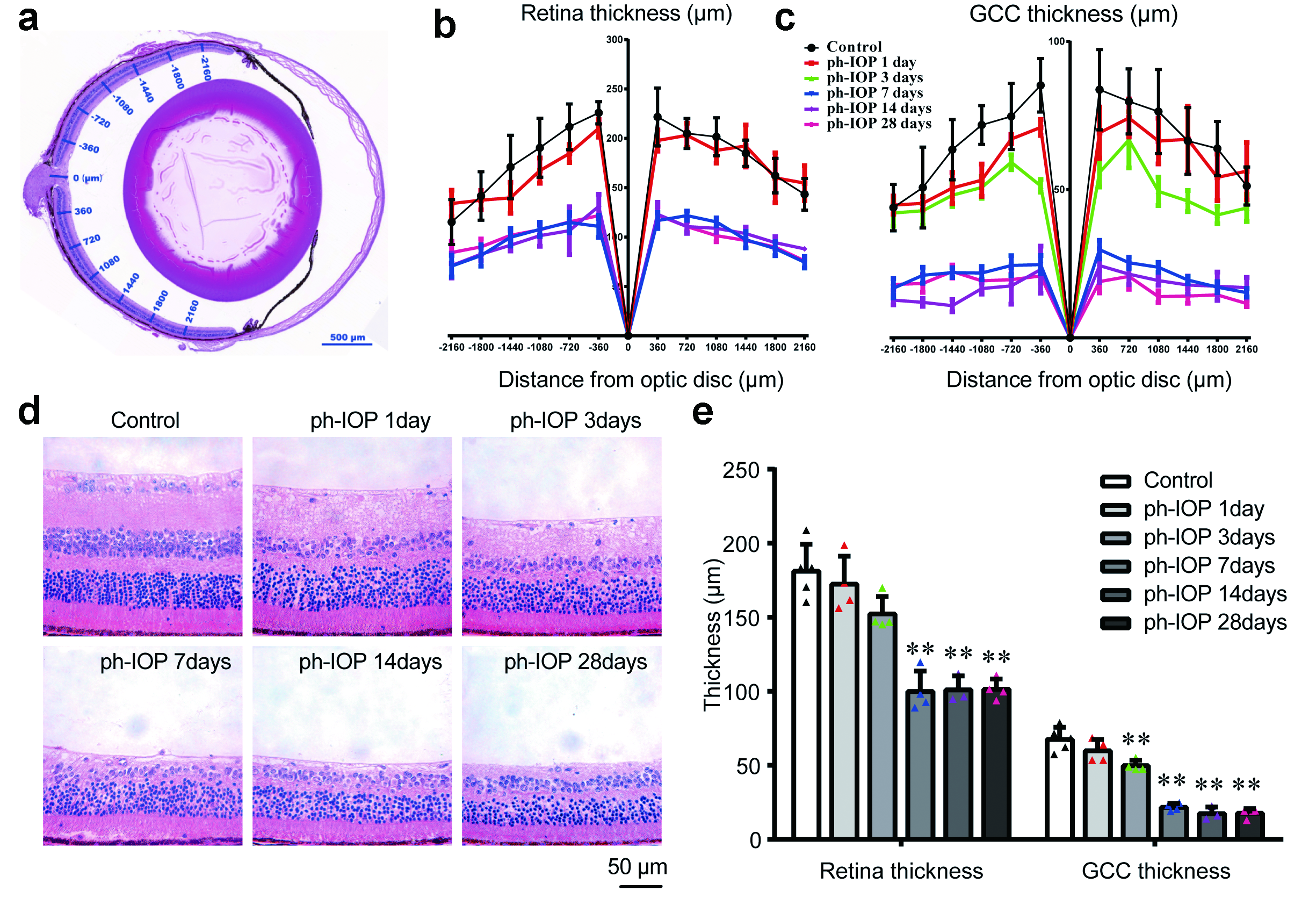

Supplement: Supplementary file 7 — Supplemental Figure 1 [file 41418_2022_1046_MOESM7_ESM.tif]

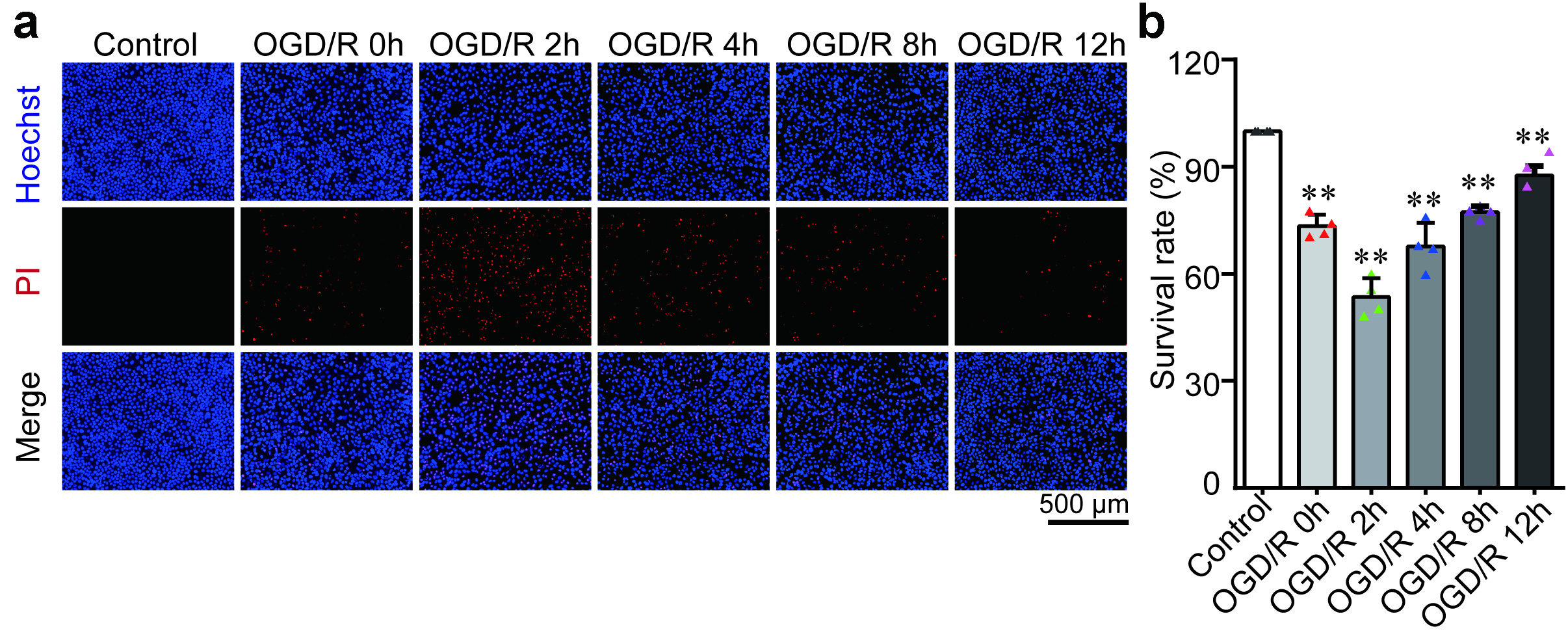

Supplement: Supplementary file 8 — Supplemental Figure 2 [file 41418_2022_1046_MOESM8_ESM.tif]

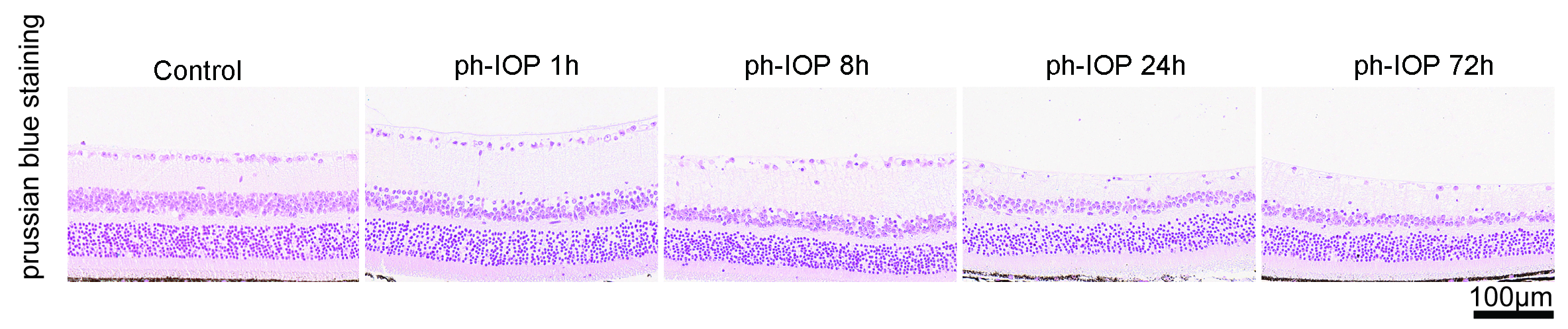

Supplement: Supplementary file 9 — Supplemental Figure 3 [file 41418_2022_1046_MOESM9_ESM.tif]

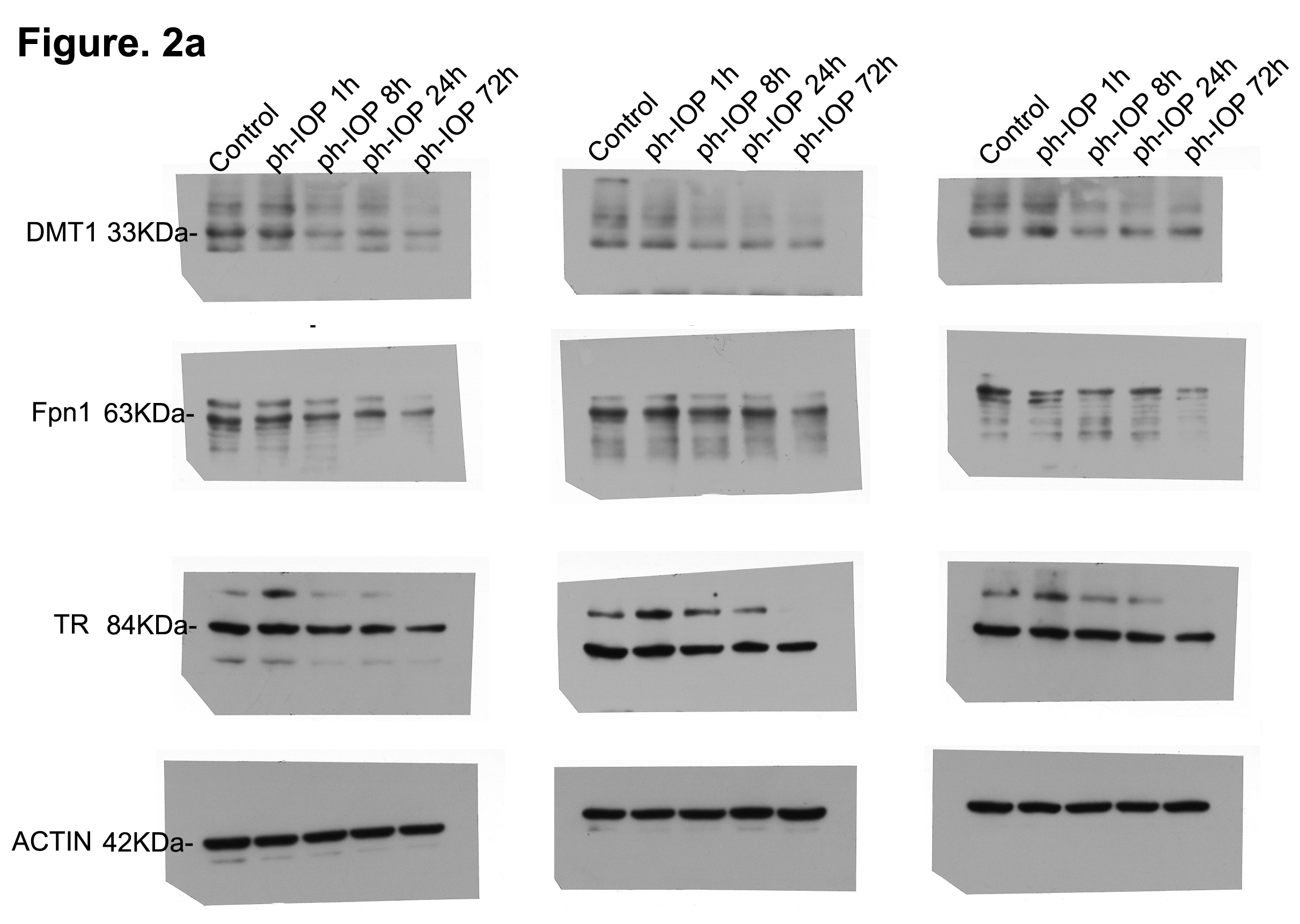


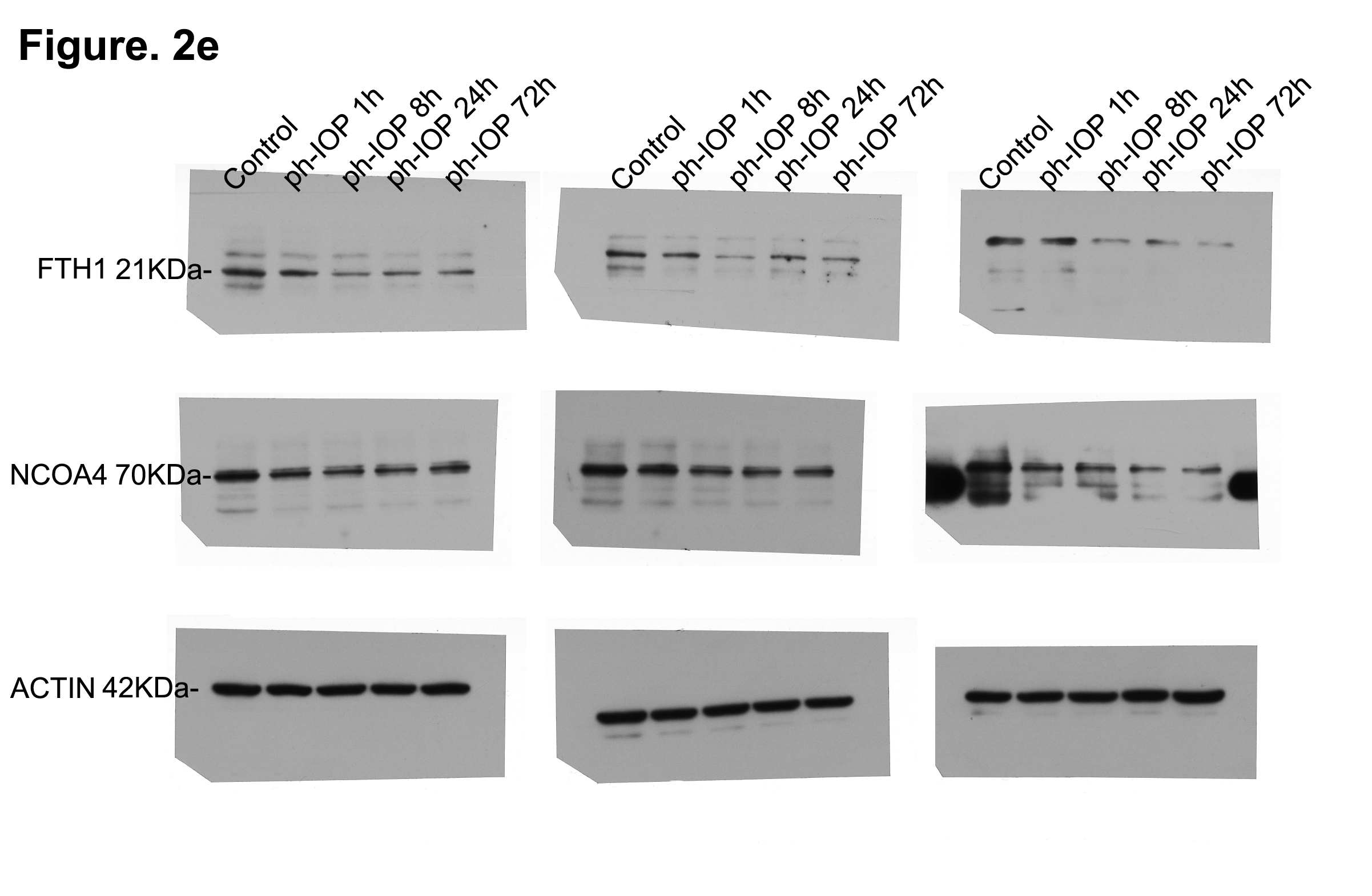

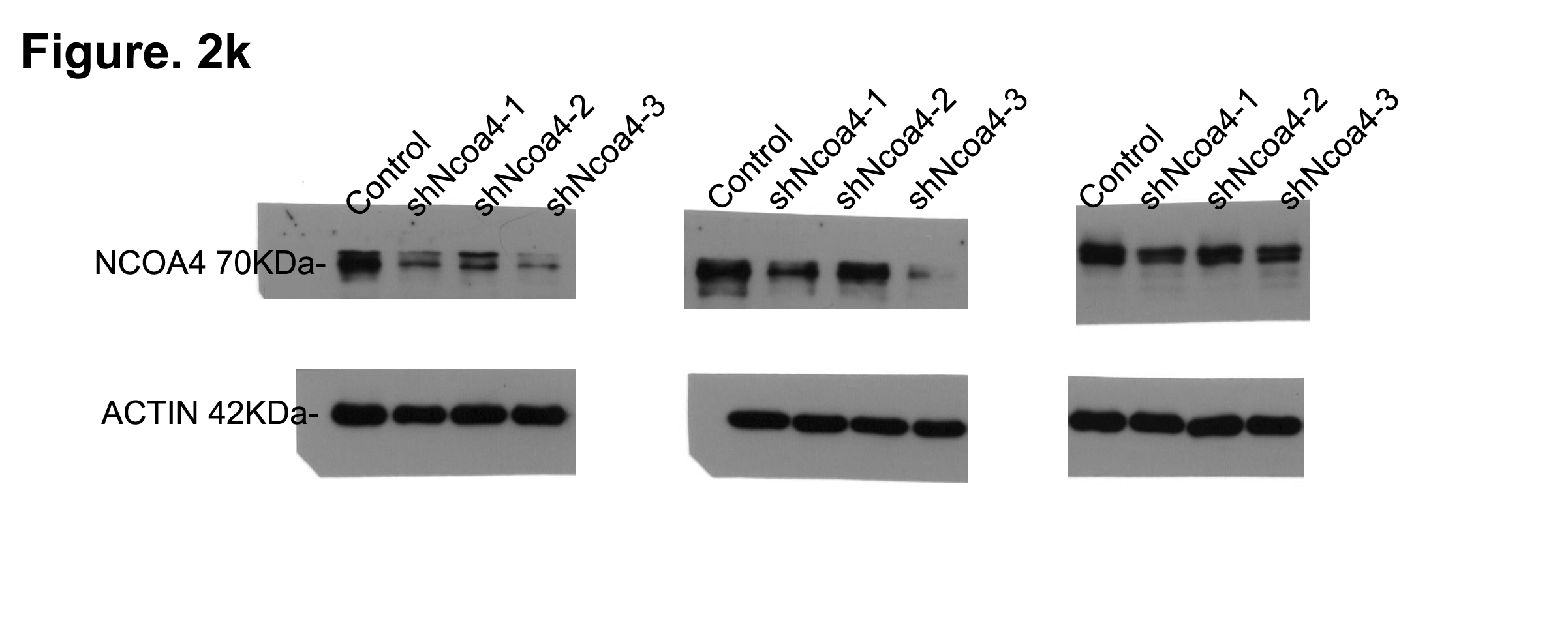

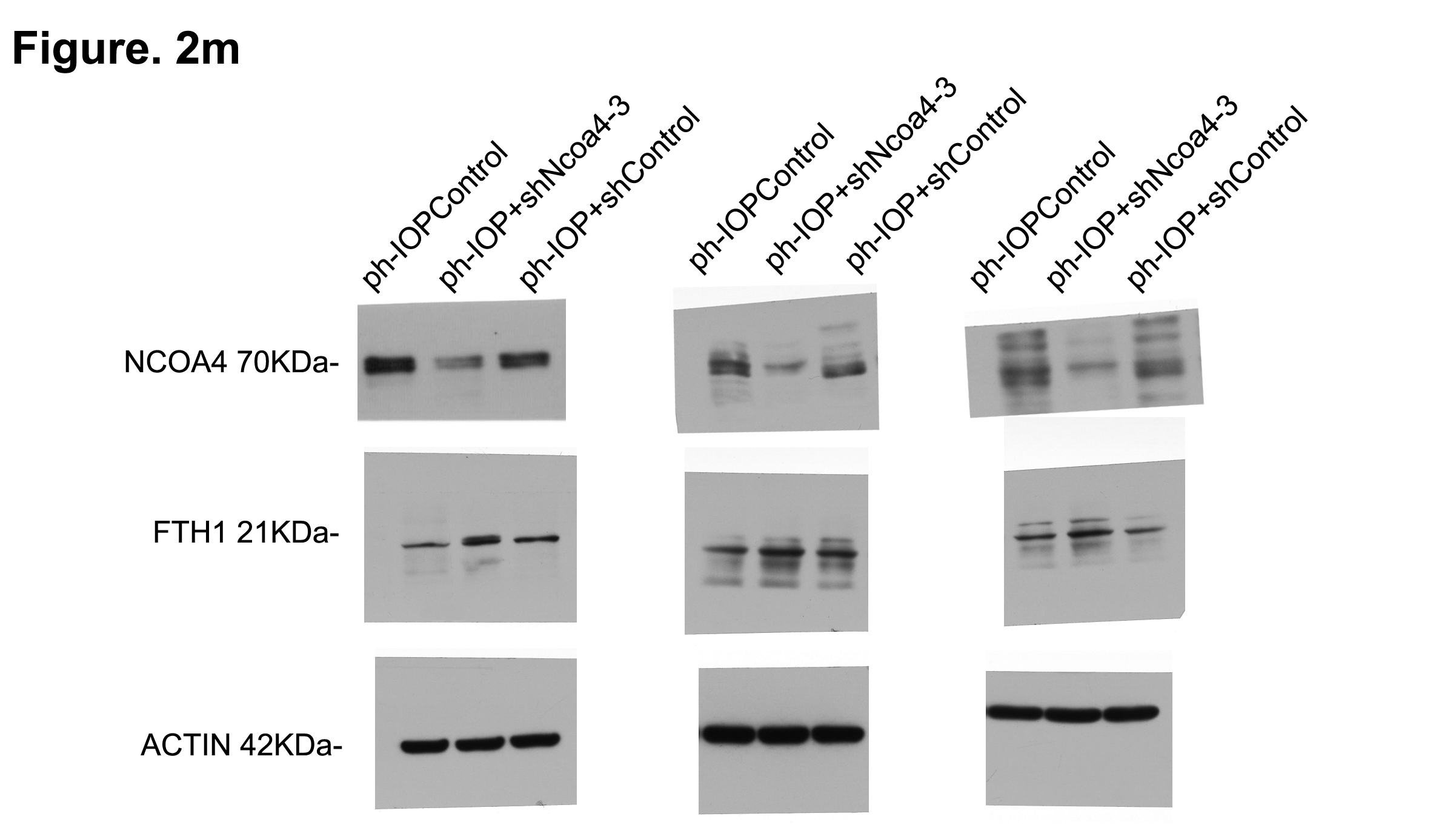

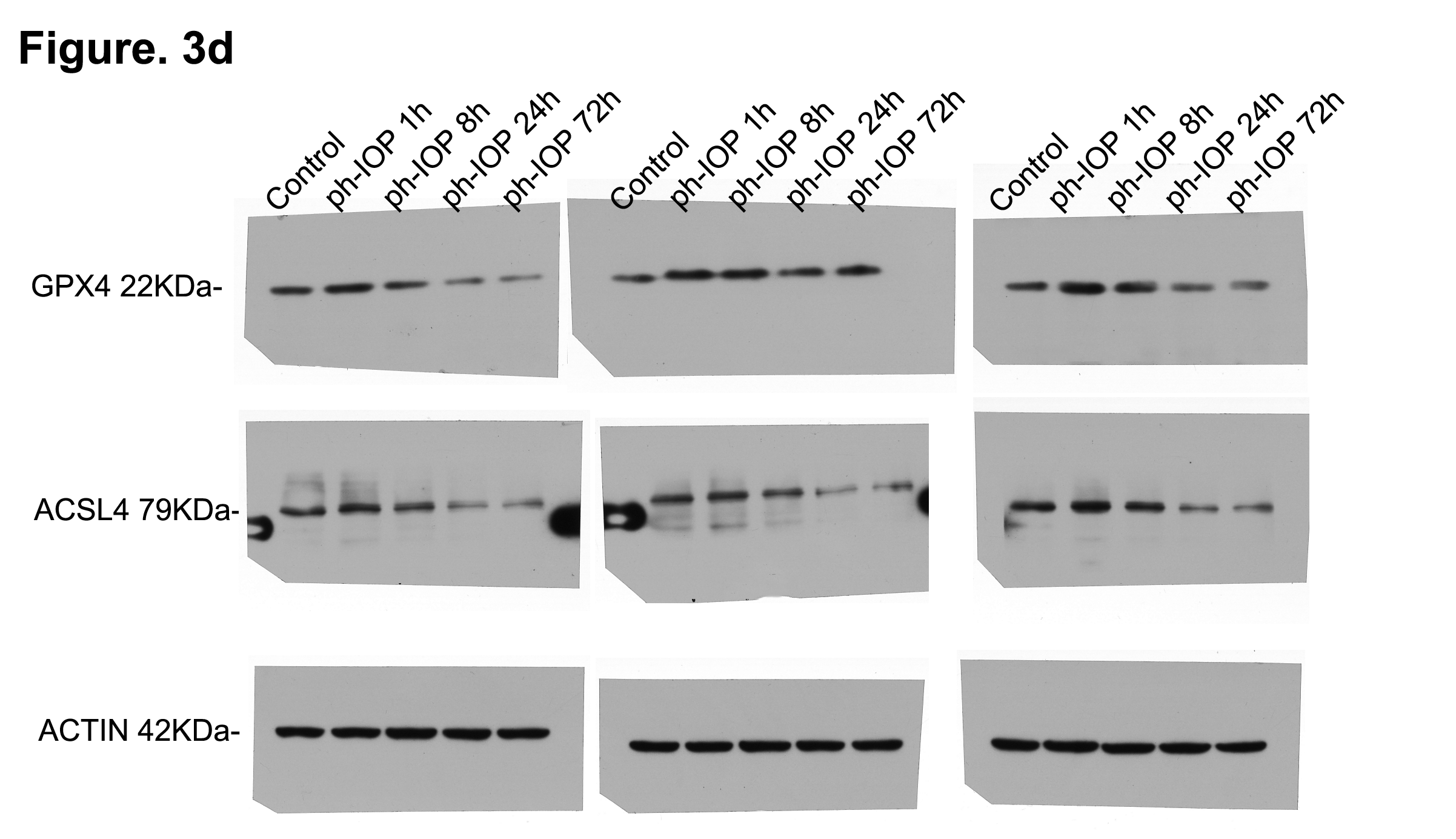

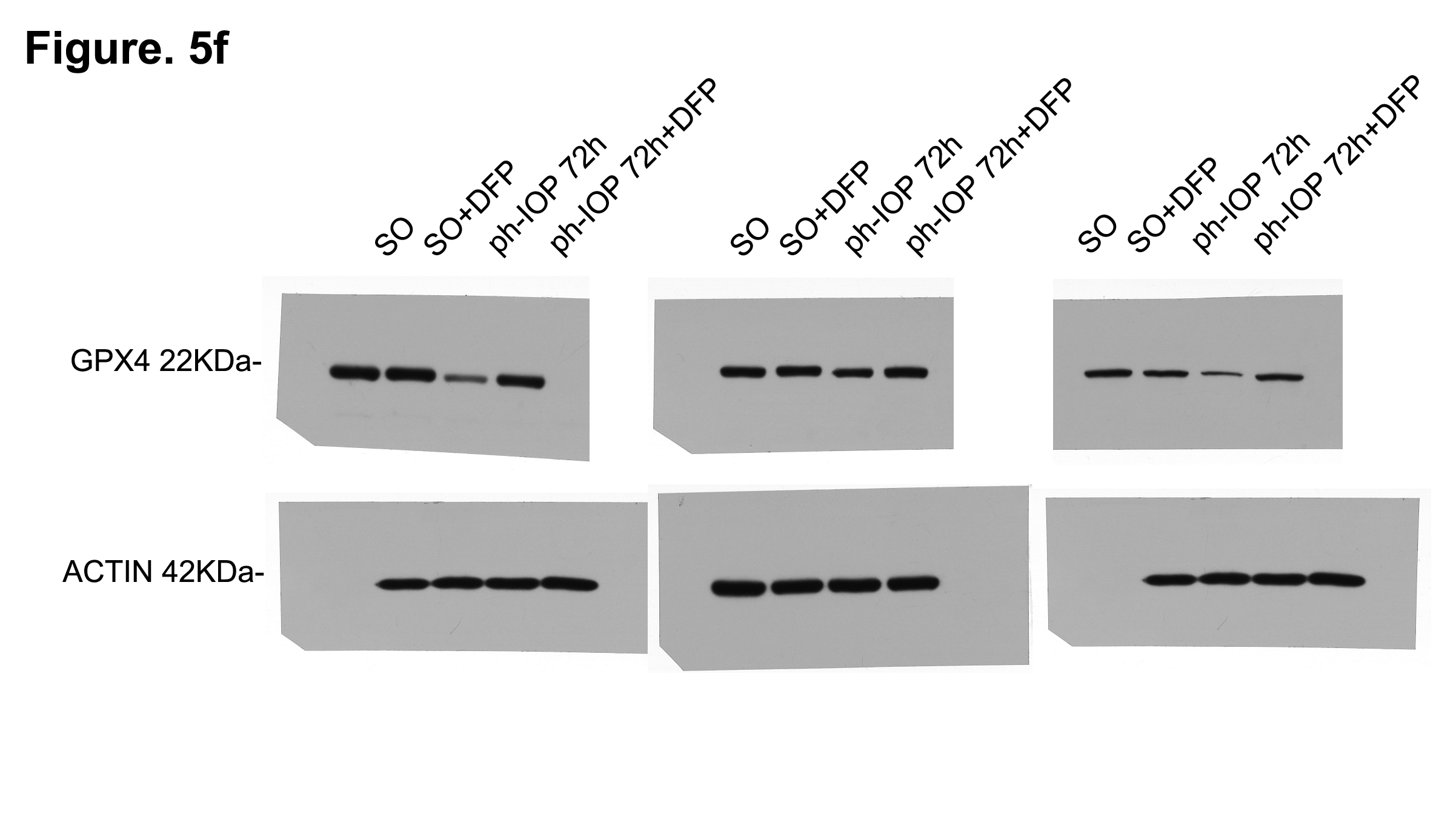

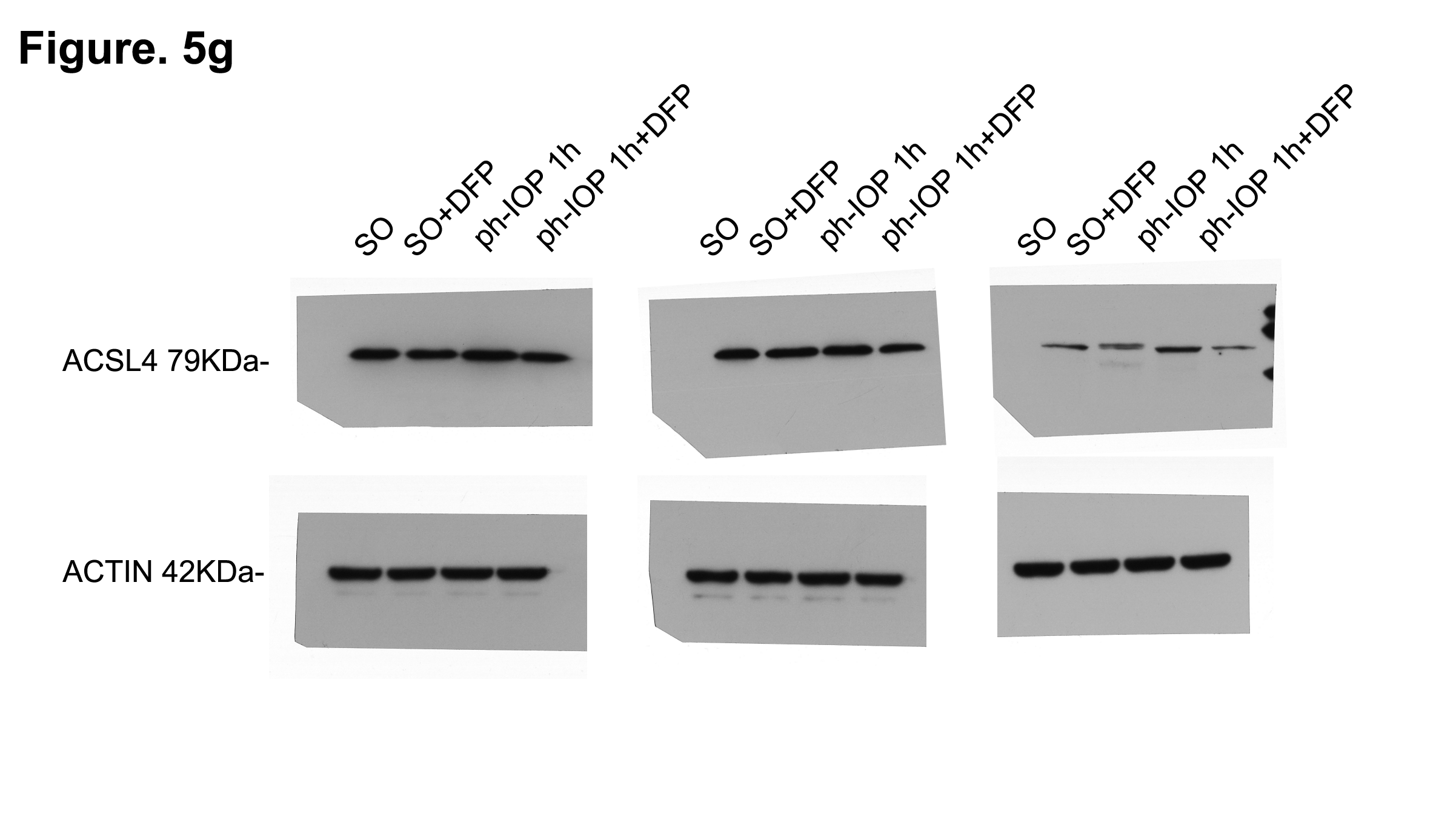

Supplement: Supplementary file 11 — Supplementary File 1 [file 41418_2022_1046_MOESM11_ESM.docx]
