# Supplementary material for: Pathologically high intraocular pressure disturbs normal iron homeostasis and leads to retinal ganglion cell ferroptosis in glaucoma
Source: Cell Death Differ. 2022 Aug 6;30(1):69–81. doi: 10.1038/s41418-022-01046-4 (PMC9883496; doi:10.1038/s41418-022-01046-4)
Supplement: Supplementary file 10 — Supplemental Figure legends [file 41418_2022_1046_MOESM10_ESM.docx]

**Supplemental Fig. S1. Retinal morphological changes after pathologically high intraocular pressure (ph-IOP) injury**. **a** Diagram of retinal thickness measurement. **b**-**c** Retina thickness and ganglion cell complex (GCC) thickness on 1, 3, 7, 14, and 28 d after ph-IOP injury. **d** Representative photomicrographs of hematoxylin and eosin (HE)-stained retinal slices after ph-IOP injury. **e** The mean thickness of the retina and GCC on 1, 3, 7, 14, and 28 d after ph-IOP modeling. Data are shown as the mean ± SD; **p < 0.01 (compared with the control group using one‑way analysis of variance). Bar = 500 μm (a) and 50 μm (d).

**Supplemental Fig. S2. The survival rate of R28 cells in the control group and oxygen-glucose deprivation/reoxygenation (OGD/R) groups at 0, 2, 4, 8, and 12 h after modeling. a** Representative photomicrographs of Hoechst and Propidium iodide (PI)-stained R28 cells at 0, 2, 4, 8, and 12 h after OGD/R modeling. **b** The survival rate of R28 cells in the control group and OGD/R groups at 0, 2, 4, 8, and 12 h after modeling. Data are shown as the mean ± SD; **p < 0.01 (compared with the control group using one-way analysis of variance). Bar = 500 μm.

**Supplemental Fig. S3. Prussian blue-stained retinal slices at 1, 8, 24, and 72 h after pathologically high intraocular pressure (ph-IOP) injury.** Bar = 100 μm.
